# ATR inhibition potentiates the antitumor efficacy of HER3-DXd in HER3-positive/HR-positive breast cancer by increasing DNA damage

**DOI:** 10.1038/s41416-026-03413-1

**Published:** 2026-04-15

**Authors:** Xuemei Xie, Jangsoon Lee, Young Jin Gi, Thanasis Poullikkas, Jon A. Fuson, Pang-Dian Fan, Jami A. Fukui, Debu Tripathy, Naoto T. Ueno

**Affiliations:** 1 Section of Translational Breast Cancer Research and The University of Texas MD Anderson Cancer Center, Houston, TX USA; 2 Department of Breast Medical Oncology, The University of Texas MD Anderson Cancer Center, Houston, TX USA; 3 Daiichi Sankyo, Inc., Basking Ridge, NJ USA; 4 Cancer Biology Program and University of Hawaiʻi Cancer Center, Honolulu, HI USA; 5 Translational and Clinical Research, University of Hawaiʻi Cancer Center, Honolulu, HI USA; 6 Present Address: Cancer Biology Program, University of Hawaiʻi Cancer Center, Honolulu, HI USA; 7 Present Address: Department of Biochemistry and Systems Biomedicine and Juntendo University Graduate School of Medicine, Bunkyo-ku, Tokyo Japan; 8 Present Address: Nakatani Biomedical Spatialomics Hub, Juntendo University Graduate School of Medicine, Bunkyo-ku, Tokyo Japan; 9 Present Address: Department of Obstetrics and Gynecology, Baylor College of Medicine, Houston, TX USA; 10 Present Address: Translational and Clinical Research Program, Clinical Trials Office, University of Hawaiʻi Cancer Center, Honolulu, HI USA

**Keywords:** Breast cancer, Translational research

## Abstract

**Background:**

Endocrine resistance remains a major challenge in hormone receptor–positive (HR+) breast cancer (BC), where up to 70% of tumours overexpress HER3, a receptor associated with poor prognosis and therapeutic resistance. HER3-DXd (patritumab deruxtecan) is currently under clinical investigation for HER3-expressing metastatic BC. However, strategies to further enhance its efficacy, particularly in endocrine therapy–resistant settings, are urgently needed. We hypothesised that targeting ATR, a key regulator of DNA damage repair (DDR), potentiates HER3-DXd in HER3+/HR+ BC, including tamoxifen–resistant (TMR) disease.

**Methods:**

Synergistic partners for HER3-DXd were identified by whole-genome RNAi screening. Treatments’ effects on cell cycle, DNA damage, and protein expression were analysed using flow cytometry, comet assay, and Western blotting, respectively. Treatments’ antitumor efficacy was assessed using xenograft mouse models. TCGA and CPTAC databases were analysed for clinical relevance.

**Results:**

HER3-DXd inhibited growth in both parental and TMR MCF7 and T47D cells. Compared to monotherapies, combining HER3-DXd with an ATR inhibitor enhanced DNA damage, sub-G1 arrest, apoptosis, downregulation of DDR and cell cycle regulatory proteins, and tumour growth inhibition. TCGA and CPTAC analyses confirmed high HER3 expression and correlation of *ATR*, *CHEK1*, and *TOP1* gene expression with poor prognosis in HR+ BC.

**Conclusion:**

Combining HER3-DXd with an ATR inhibitor could benefit HER3+/HR+ BC patients with both endocrine–sensitive and –resistant diseases.

## Introduction

The epidermal growth factor receptor tyrosine kinases (ErbBs), including ErbB1 (also known as EGFR and HER1), ErbB2 (HER2), ErbB3 (HER3), and ErbB4 (HER4), play critical roles in cancer development and progression [1–3]. While EGFR and HER2 have long been used as therapeutic targets for cancer treatment [4, 5], HER3 has recently emerged as a critical driver of tumorigenesis and metastasis. Despite its weak intrinsic kinase activity, HER3 promotes these processes by activating downstream signalling through heterodimerization with other ErbB family members and other receptor tyrosine kinases [6]. Thus, targeting HER3 can potentially suppress downstream signalling by disrupting its interaction with heterodimeric partners [6], holding promise for therapeutic intervention.

Approximately two-thirds of breast cancers (BCs) are hormone receptor–positive (HR+ ), expressing oestrogen receptor (ER) and/or progesterone receptor (PR) [7], and are typically treated with endocrine therapies. These include selective ER modulators (e.g., tamoxifen), aromatase inhibitors (e.g., letrozole, anastrozole, and exemestane), and ovarian suppression (i.e., via gonadotropin-releasing hormone agonists or surgery) [8]. Other therapies, most used in the metastatic setting, include CDK4/6 inhibitors (e.g., ribociclib and palbociclib), selective ER degraders (e.g., fulvestrant), and mTOR inhibitors (e.g., everolimus) [8]. Although endocrine therapies have improved outcomes in patients with HR+ BC, both intrinsic and acquired resistance remain major clinical challenges. Approximately 25% to 30% of patients do not respond to initial endocrine therapy, and many eventually develop resistance [9, 10], underscoring the urgent need for new treatment strategies for patients with HR+ BC.

HER3 is overexpressed in up to 70% of HR+ BCs and is associated with poor outcomes and resistance to therapy, making it a compelling target. HER3-DXd (patritumab deruxtecan; also known as U3-1402) is a first-in-class HER3-directed antibody-drug conjugate (ADC) composed of a humanised anti–human HER3 IgG1 antibody (patritumab) linked to a TOP1 inhibitor (DXd) via a cleavable tetrapeptide linker, with a drug-to-antibody ratio of approximately 8 [11]. HER3-DXd has demonstrated an acceptable safety profile and promising antitumor efficacy in clinical trials involving heavily pretreated HER3+ BC patients, including those with HR+/HER2- BC (NCT02980341, NCT04699630, and NCT04965766) [12–14]. It also showed substantial central nervous system (CNS) activity in patients with brain metastases and leptomeningeal disease (NCT05865990) [15], highlighting its potential utility for treating broader CNS disease in cancer patients.

Ataxia-telangiectasia and Rad3-related protein (ATR) is a key regulator of the DNA damage repair (DDR) activated by single-strand DNA breaks, DNA damage, and DNA replication stress [16, 17]. Once activated, ATR phosphorylates and activates Chk1 and other substrates to ensure genome integrity by halting cell cycle progression and facilitating DDR, subsequently promoting cell survival [16, 17]. Homozygous ATR inactivation is embryonically lethal in mice [18]. ATR inhibitors were selectively cytotoxic to cancer cells with high replication stress and robustly synergised with nearly all tested genotoxic therapies [17]. These studies demonstrate the importance of ATR in cancer cell survival and highlight the potential of targeting ATR for cancer treatment.

In this study, we investigated whether combining HER3-DXd with the ATR inhibitor BAY 1895344 could enhance antitumor activity in endocrine therapy–resistant HER3+/HR+ BC models. We found that compared with HER3-DXd and BAY 1895344 monotherapies, the combination treatment exhibited greater growth-inhibiting activity against tamoxifen-resistant (TMR) MCF7 and T47D BC cells both in vitro and in vivo. Mechanistic studies showed that the combination treatment synergistically reduced expression of key ATR signalling proteins, including TOPBP1 (topoisomerase II binding protein 1 and ATRIP (ATR-interacting protein), and suppressed ATR activation. ATR inactivation impaired DDR, increased DNA damage accumulation, and induced cell cycle arrest via inhibition of Chk1/cyclin A1/CDK2 and Chk1/cyclin E/CDK2 signalling, eventually leading to apoptotic cell death. Our findings demonstrate the therapeutic potential of combining HER3-DXd with an ATR inhibitor to overcome endocrine therapy resistance in HER3+/HR+ BC, providing a strong rationale for further clinical development of this strategy.

## Materials and methods

### Analysis of HER3 gene and protein expression in BC subtypes

The Cancer Genome Atlas (TCGA) Breast Invasive Carcinoma dataset was accessed through the UALCAN web portal (http://ualcan.path.uab.edu; accessed on August 24, 2024) and used to compare *ERBB3* mRNA expression levels between normal breast tissue and invasive BC subtypes, including luminal/ER+, HER2+, and triple-negative BC (TNBC). The Clinical Proteomic Tumour Analysis Consortium (CPTAC) dataset was also accessed through UALCAN and used to compare HER3 protein expression levels between normal tissues and invasive BC subtypes. The TCGA mRNA and CPTAC protein datasets represent distinct patient cohorts and were analysed independently within the UALCAN platform. All analyses were performed using UALCAN’s built-in analytical tools [19].

### Analysis of prognostic impact of target gene expression in HR+ and overall BC

The prognostic impact of target gene expression was evaluated using the Kaplan–Meier Plotter online tool (http://kmplot.com; accessed on September 7, 2025), which integrates multiple public BC microarray datasets and allows stratification by clinical subgroups [20]. Recurrence-free survival (RFS) and distant metastasis–free survival (DMFS) were analysed in two cohorts: (1) an HR+ BC cohort treated with endocrine therapy alone or in combination with chemotherapy (ER-positive [ER+], PR-positive [PR], HER2-negative; *n* = 464 for RFS and *n* = 383 for DMFS) and (2) an overall BC cohort, including all clinical subtypes (maximum *n* = 4926 for RFS and *n* = 2765 for DMFS). These patient datasets are distinct from the TCGA dataset described above for HER3 gene expression. Survival curves were generated by stratifying patients into “High” vs “Low” expression groups based on the optimal cutoff value determined automatically by the Kaplan–Meier Plotter software, which also calculated hazard ratios and log-rank *P* values.

Because the HR+ and overall BC analyses included different patient populations and array platforms, different Affymetrix probe IDs were used for some genes in each dataset. The optimal probe for each gene was selected based on availability and performance (JetSet best probe) within each cohort. Specifically, the following probes were used; when two probes are listed for the same gene, the first probe was used in the HR+ dataset and the second probe was used in the overall BC dataset: *TOP1*, 208900_s_at; *TOPBP1*, 202633_at; *ATR*, 208531_at; *ATRIP*, 34689_at and 1552937_s_at; *CHEK1*, 205394_at and 238075_at; *H2AFX*, 205436_s_at; *CDK2*, 204252_at; *CDK3*, 207188_at; *CDK6*, 207143_at and 224847_at; *CCNA1*, 213226_at; *CCNB1*, 214710_s_at and 228729_at; *CCNC*, 201955_at; and *CCNE1*, 213523_at.

All survival analyses were performed using the “Single gene analysis” function of Kaplan–Meier Plotter with “Auto select best cutoff” for grouping and the above probe set selections. For the HR+ BC cohort, the analyses were restricted to ER+, PR+, HER2-negative tumours from patients who received endocrine therapy with or without chemotherapy, whereas for the overall BC cohort, all subtypes were included. The number of patients in each “High” and “Low” expression group is indicated on the respective survival plots.

### Cell lines and reagents

MCF7, T47D, MDA-MB-175, MDA-MB-134, and HCC1500 HER3+/ER+ BC cells and MDA-MB-231 HER3-negative TNBC (ER, PR, and HER2 negative) cells were purchased from American Type Culture Collection (Manassas, VA, USA). BCX-010 HER3-negative TNBC cells were a generous gift from Dr. Funda Meric-Bernstam (MD Anderson Cancer Center, Houston, TX, USA). TMR MCF7 (MCF7-TMR; #SCC101) and TMR T47D (T47D-TMR; #CB_16022513) cells were purchased from Sigma-Aldrich (St. Louis, MO, USA).

MCF7 and MCF7-TMR HER3+/HR+ cells were cultured in Dulbecco modified Eagle medium/F12 medium without Phenol Red (#D6434; Sigma-Aldrich) supplemented with 1% foetal bovine serum (FBS; #F0600-050; GenDEPOT, Katy, TX, USA), 1% antibiotic/antimycotic (#A5955; Sigma-Aldrich), 2.5 mM Ala-Gln (#G8541; Sigma-Aldrich), and 1 µg/mL insulin (#12-585-014; Thermo Fisher Scientific, Waltham, MA, USA). T47D and T47D-TMR cells were cultured in RPMI 1640 medium without Phenol Red (#R7509; Sigma-Aldrich) supplemented with 2% FBS, 1% antibiotic/antimycotic, 2.5 mM Ala-Gln, and 8 µg/mL insulin. Per the manufacturer’s documentation, the TMR derivatives (MCF-TMR and T47D-TMR) were established by long-term exposure to tamoxifen (up to 1 μM) under hormone-deprived conditions (2% FBS). To maintain their resistant phenotype, both MCF7-TMR and T47D-TMR cells were routinely cultured in the presence of 1 μM tamoxifen. Their resistant phenotype was further confirmed under our experimental conditions prior to use in our study. MDA-MB-175 and MDA-MB-231 cells were cultured in Dulbecco modified Eagle medium/F12 medium (#D8062; Sigma-Aldrich) supplemented with 10% FBS and 1% antibiotic/antimycotic. BCX-010 cells were cultured in Ham’s F-12 medium supplemented with 10% FBS, 1 µg/mL hydrocortisone (#H0888, Sigma-Aldrich), 5 µg/mL insulin, and 1% antibiotic-antimycotic. HCC1500 cells were maintained in RPMI 1640 medium (#R8758; Sigma-Aldrich) supplemented with 10% FBS and 1% antibiotic/antimycotic.

All cell lines used in this study were validated by the Cytogenetics and Cell Authentication Core Facility at MD Anderson Cancer Center using a short tandem repeat method based on a primer extension to detect single-base deviations and were confirmed to be free of *Mycoplasma* using the MycoAlert *Mycoplasma* Detection Kit (#LT07-710; Lonza, Morristown, NJ, USA).

HER3-DXd, control IgG-DXd, and patritumab were provided by Daiichi Sankyo Inc. (Basking Ridge, NJ, USA). ATR inhibitors AZD6738 (#S7693) and BAY 1895344 (#S9864), ROCK2 inhibitor azaindole 1 (#S6636), RAB7A inhibitor CID1067700 (#E0135), and SLC29A1/ENT1 inhibitor NBMPR (S-(4-nitrobenzyl)-6-thioinosine; #S0838) were purchased from Selleck Chemicals LLC (Houston, TX, USA).

### Soft agar colony formation assay

To test the growth-inhibiting effects of HER3-DXd alone or combined with small molecular inhibitors, cells (5 × 10^3^ cells/well) were suspended in 0.375% agarose (#16520050; Thermo Fisher Scientific) in a complete medium containing HER3-DXd (5 nM) alone or combined with ATR inhibitor AZD6738 (0.1 and 0.5 µM) or BAY 1895344 (0.005, 0.01, 0.025, or 0.05 µM), ROCK2 inhibitor azaindole 1 (0.5 and 0.5 µM), RAB7A inhibitor CID1067700 (0.5 and 1 µM), or SLC29A1/ENT1 inhibitor NBMPR (0.5 and 1 µM) in 12-well plates. For the HER3-DXd and BAY 1895344 combination, IgG-DXd (5 nM) or patritumab (5 nM) combined with BAY 1895344 (0.005, 0.01, 0.025, or 0.05 µM) was used as a control. The mixtures were overlaid onto a 0.75% agarose layer in 12-well plates, and the plates were incubated at 37 °C with 5% CO_2_ for up to 3 weeks. After incubation, the colonies were stained with MTT (3-(4,5-dimethylthiazol-2-yl)-2,5-diphenyltetrazolium bromide; #M5655; Sigma-Aldrich) for 2 h at 37 °C. Colonies larger than 80 μm in diameter were counted using a GelCount system (Oxford Optronix, Milton, UK).

### Clonogenic assay

To test the growth-inhibiting effects of HER3-DXd alone or combined with BAY 1895344, cells (2–5 × 10^4^ cells/well) were seeded in 6-well plates, cultured overnight at 37 °C, and treated the next day with HER3-DXd (10 nM), BAY 1895344 (0.005, 0.01, 0.025, or 0.05 µM), or HER3-DXd plus BAY 1895344. Cells treated with BAY 1895344 plus IgG-DXd (10 nM) or patritumab (10 nM) were used as the controls. The plates were cultured at 37 °C for up to 3 weeks, and the media containing drugs were replenished weekly. To test the growth-inhibiting effects of HER3-DXd alone or combined with siRNA targeting ATR or TOP1, cells were transfected with siRNA (5 µM) and then seeded in 6-well plates 24 h after transfection. The cells were treated the next day with HER3-DXd (10 nM) or IgG-DXd (10 nM) for up to 3 weeks. After treatment, the colonies were fixed with 5% trichloroacetic acid (#T8657; Sigma-Aldrich) and then stained with 0.03% sulforhodamine B solution (#230162; Sigma-Aldrich) for 30 min at room temperature. Colonies larger than 80 µm in diameter were counted using the GelCount system.

### High-throughput RNAi screening

To identify genes whose inhibition might enhance the antitumor efficacy of HER3-DXd, we performed high-throughput RNAi screening using MCF7 cells, which express high levels of HER3 and are well characterised in preclinical models. We used Ambio *Silencer* Select Human Genome siRNA Library V4 (#4397926; Life Technologies, Carlsbad, CA, USA), consisting of 64,752 siRNAs targeting 21,584 genes, with 3 unique siRNAs targeting each gene. MCF7 cells were transfected with the siRNA library using a reverse transfection procedure. In brief, siRNAs (2 µM) were seeded in 384-well plates, and then lipofectamine RNAiMAX (Invitrogen, Waltham, MA, USA) and MCF7 cells (3000 cells/well) were added. The mixtures were incubated for 24 h and then treated with HER3-DXd at IC_20_ (20 nM) for 5 days at 37 °C. After 5-day treatment, ATPlite 1step (#6016739; PerkinElmer, Waltham, MA, USA) was added, and the cultures were incubated for 10 minutes at room temperature. Luminescence readings of each treatment were normalised to the mean of the no-siRNA negative control in the same plate. As previously described [21, 22], the sensitivity index was calculated for each gene after treatment with siRNA and HER3-DXd and was used to identify genes whose inhibition might be synergistic with HER3-DXd.

### Cell cycle and apoptosis analyses

Cells (5 × 10^5^ cells) were seeded in 60-mm plates and cultured overnight. The cells were treated the next day with HER3-DXd (10 nM), BAY 1895344 (0.01 µM), or HER3-DXd plus BAY 1895344 for 72 h at 37 °C. After 72-h treatment, the cells were collected and used for cell cycle and apoptosis analyses. For the cell cycle analysis, the cells were fixed in ice-cold 70% EtOH (#E7023; Sigma-Aldrich), treated with 50 μg/mL RNase A (#12091021; Invitrogen), stained with propidium iodide (#P4864; Sigma-Aldrich), and then analysed by flow cytometry. For the apoptosis analysis, the cells were incubated with 7-amino-actinomycin and Annexin V- phycoerythrin (#640934; BioLegend, San Diego, CA, USA) and then analysed for apoptosis by flow cytometry.

### Alkaline comet assay

The alkaline comet assay, a common assay to detect both single-strand and double-strand DNA breaks in cells, was used to quantify DNA fragmentation. Cells (1 × 10^6^ cells) were seeded in 10-cm plates overnight and were treated the next day with HER3-DXd (10 nM), BAY 1895344 (0.01 µM), or HER3-DXd plus BAY 1895344 for 48 h at 37 °C. After treatment, cells were collected and resuspended in 30 μL of complete medium (2 × 105 cells) and then mixed with 300 μL of Comet LMAgarose (#4250-050-02; R&D Systems, Minneapolis, MN, USA). The mixture was added to each well of a 3-well CometSlide (#4250-050-03; R&D Systems) and incubated at 4 °C for 15 min. To increase cell membrane permeability, slides were immersed in 50 mL of a pre-chilled lysis solution (#4250-050-01; R&D Systems) for 30 min at 4 °C and in 50 mL of alkaline unwinding solution for 20 min at room temperature in the dark. Following the unwinding procedure, electrophoresis was performed at 4 °C. The slides were fixed in 70% EtOH, stained with SYBR Gold Nucleic Acid Gel Stain solution (#S11494; Thermo Fisher Scientific) for 30 min at room temperature in the dark, and dried at 37 °C. Images were captured using a Keyence confocal microscope (VK-X3000; Keyence Corporation; Osaka, Japan), and comet formation was measured using the ImageJ software plug-in OpenComet (National Institutes of Health).

### Western blot analysis

The effects of treatments on target protein expression were analysed using Western blotting as described previously [23]. Proteins of interest on the blots were probed using the following primary antibodies (at 1:1000 dilution), which were purchased from Cell Signalling Technology or other suppliers as indicated: anti-HER3 (#12708), anti-ATR (#2790), anti-phospho-ATR (pATR) at Ser428 (#2853), anti-pATR at Thr1989 (#58014), anti-ATRIP (#2737), anti-TOP1 (#38650), anti-TOPBP1 (#14342), anti-Chk1 (#25887-1-AP; Proteintech, Rosemont, IL, USA), anti-phospho-Chk1 (pChk1) at Ser296 (#2349), anti-H2AX (ab124781; Abcam, Cambridge, United Kingdom), anti-γH3AX at Ser139 (#ab81299; Abcam), anti-CDK1 (#28439), anti-phospho-CDK1 at Tyr15 (#4539), anti-CDK2 (#18048), anti-phspho-CDK2 at Thr160 (#2561), anti-CDK3 (#ab96847; Abcam), anti-CDK6 (#ab124821; Abcam), anti-cyclin A2 (#ab211735, Abcam), anti-cyclin B1 (#12231), anti-cyclin C (#ab85927; Abcam), anti-cyclin E1 (#20808), PARP (#9532), cleaved PARP (#5625), and β-actin (#A5316; Sigma-Aldrich). The secondary antibody was horseradish peroxidase-conjugated IgG (1:10,000 dilution; Life Technologies) for chemiluminescent signal detection.

### MCF7-TMR and T47D-TMR xenograft models

MCF7-TMR or T47D-TMR cells (4 × 10^6^ cells/100 µL/mouse) in log-phase growth were resuspended in complete medium containing 50% Matrigel and then injected under aseptic conditions into the mammary fat pads of 4–6-week-old female NSG mice (NOD.Cg-Prkdc^scid Il2rg^tm1Wjl/SzJ; stock no. 005557; The Jackson Laboratory, Bar Harbour, ME, USA). When tumour size reached 300 mm³ for the MCF7-TMR model and 230 mm³ for the T47D-TMR model, mice were randomly divided into four groups (10 mice/group) and treated with IgG-DXd (1 mg/kg, weekly, intravenously), HER3-DXd (1 mg/kg, weekly, intravenously), BAY 1895344 (7.5 mg/kg, 3 days/week, orally), or HER3-DXd plus BAY 1895344 for 25 days for the MCF7-TMR model and 45 days for the T47D-TMR model. Tumour size and mouse body weight were measured biweekly. Animal studies were approved by the Institutional Animal Care and Use Committee of MD Anderson Cancer Center (protocol 00001754-RN01). Animal care and experiments were conducted per Institutional and National Institutes of Health guidelines. Tumour tissues were collected on day 28 from mice bearing MCF7-TMR xenografts and on day 45 from mice bearing T47D-TMR xenografts following treatments and processed for immunohistochemistry staining. No animals or tumour samples were excluded from the analysis, and no additional pre-specified inclusion or exclusion criteria were applied beyond the general health and eligibility criteria. Investigators were not blinded to group allocation during treatment administration or tumour volume measurement. Outcome assessment was performed with knowledge of the assigned treatment groups. No additional blinding procedures were implemented.

### Immunohistochemical staining

Tumour tissues were collected on day 28 from mice bearing MCF7-TMR xenografts and on day 45 from mice bearing T47D-TMR xenografts following treatments. Formalin-fixed, paraffin-embedded sample blocks were prepared and processed for immunohistochemistry staining as previously described [24]. The proteins of interest on tumour tissues were detected using the following antibodies purchased from Cell Signalling Technology or other suppliers as indicated: anti-HER3 (#12708), anti-pATR at Ser428 (#ab178407; Abcam), anti-γH3AX at Ser139 (#ab81299; Abcam), and cleaved PARP (#5625). Expression levels of proteins were measured using ImageJ software (National Institutes of Health). Images were captured at 20x magnification using an Eclipse 80i microscope (Nikon Instruments, Melville, NY, USA).

### Statistical analysis

Data were summarised with descriptive statistics (mean ± standard deviation [SD]). Clinical endpoints included RFS and DMFS, both measured from the date of diagnosis. Time-to-event endpoints were estimated using the Kaplan–Meier method. All computations were carried out using SAS 9.3 (SAS Institute Inc, Cary, NC, USA). Statistical analysis of all in vitro data was performed by using two-tailed unpaired Student’s *t*-tests in GraphPad Prism version 10. Drug combination effects were evaluated with CalcuSyn version 2.0 to calculate the combination index (CI) and fraction affected (Fa), which were used to evaluate the synergistic effect of combination treatment. For in vivo tumour growth studies, once tumours reached the target baseline volume, mice were allocated to treatment groups by simple randomisation to achieve comparable mean baseline tumour sizes across groups. Sample size (10 mice per group) was determined a priori based on power calculations from a similar prior xenograft experiment, assuming a 30% reduction in mean tumour volume between treatment and control, a SD of approximately 0.3 on the analysis scale, and a multiple-comparison–adjusted α = 0.0083 (0.05/6) for pairwise comparisons among four treatment groups at a single time point, providing ~77% power for a two-sided comparison. Differences in tumour volumes between treatment groups were determined using the Mann–Whitney U test. A two-sided *P* ≤ 0.05 was considered statistically significant.

## Results

### HER3 expression level was higher in ER+ BC than in other subtypes

**Fig. 1:**
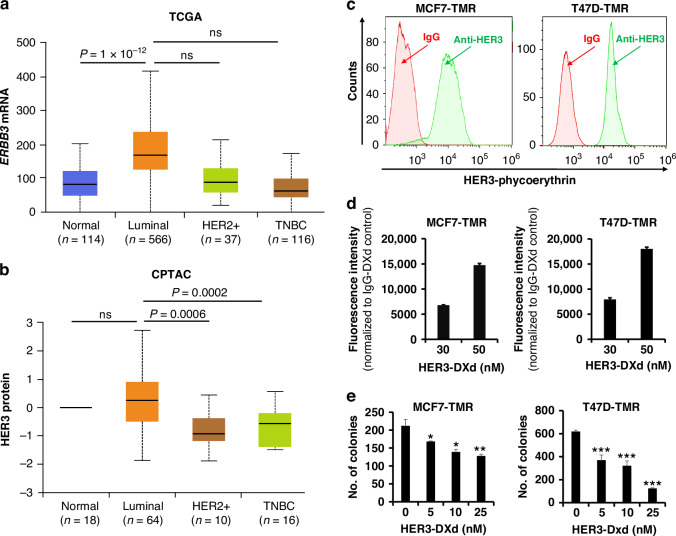
HER3 expression in human breast tumours, HER3-DXd internalisation into HER3+/HR+ TMR MCF7 and T47D BC cells, and growth-inhibiting activity of HER3-DXd against HER3+/HR+ TMR MCF7 and T47D BC cells. Expression of HER3 at (**a**) mRNA and (**b**) protein levels in normal human breast tissues and subtypes of human breast tumours from analysis of TCGA and CPTAC datasets, respectively. ns: not significant. **c** HER3 expression on the surface of MCF7-TMR and T47D-TMR cells as determined by flow cytometry. **d** Internalisation of HER3-DXd into MCF7-TMR and T47D-TMR cells as measured using pHAb amine reactive dye. **e** HER3-DXd inhibits colony formation as determined by clonogenic assay in MCF7-TMR and T47D-TMR cells. Bars show the mean ± SD. *P < 0.05, ** *P* < 0.01, *** P < 0.001, vs. the untreated control. Statistical significance was analysed by two-tailed unpaired Student’s *t*-tests. In **c** and **d**, the data are representative of triplicates from one of two independent experiments. In **e**, the data are representative of triplicates from one of three independent experiments.

To identify BC patients who may benefit from HER3-DXd, we analysed *ERBB3* mRNA levels across BC subtypes using the TCGA dataset. Luminal breast tumours, the majority of which are ER+, expressed higher levels of *ERBB3* (median expression level [MEL] = 168.13) than normal breast tissues (MEL = 82.64), HER2+ BC (MEL = 89.06), and TNBC (MEL = 62.62) (Fig. 1a). The CPTAC dataset analysis confirmed that HER3 protein levels were also higher in luminal breast tumours than in HER2+ BC and TNBC (Fig. 1b). These results demonstrate that HER3 expression levels are higher in ER+ BC than in other BC subtypes. On the basis of these findings, we tested the antitumor efficacy of HER3-DXd using HER3+/HR+ BC preclinical models.

### HER3-DXd’s growth-inhibiting activity depended on its binding to HER3

In current clinical practice, the majority of patients with HR+ metastatic BC receive endocrine therapy. HER3 is involved in resistance to endocrine therapy (e.g., tamoxifen) [25, 26]. Therefore, we used MCF7-TMR and T47D-TMR cells as endocrine therapy–resistant models and assessed whether HER3-DXd could overcome resistance to endocrine therapy in BC. We also used those two models, along with their parental MCF7 and T47D cells to identify genes whose inhibition might enhance the antitumor efficacy of HER3-DXd and to explore the downstream molecular mechanism underlying the enhancement.

Before testing the growth-inhibiting effect of HER3-DXd, we assessed its internalisation into HER3+/HR+ BC cells that express high levels of HER3 (Figs. 1c, S1a) using pHAb amine reactive dye. HER3-DXd was effectively internalised into HER3+/HR+ BC cells (Fig. 1d, S1a), suggesting that HER3-DXd can effectively deliver DXd into HER3+/HR+ BC cells. No significant difference was found in HER3-DXd internalisation between MCF7 and MCF7-TMR cells or between T47D and T47D-TMR cells (Figs. 1d, S1b). This may be due to the similar surface expression levels of HER3 on MCF7 and MCF7-TMR cells and on T47D and T47D-TMR cells.

We next evaluated the growth-inhibiting activity of HER3-DXd as a single agent in vitro. HER3-DXd at nanomolar concentrations effectively inhibited the growth of MCF7-TMR, T47D-TMR, MCF7, T47D, HCC1500, and MDA-MB-175 cells, as determined by clonogenic assay (Figs. 1e, S1c). The sensitivity of MCF7-TMR and T47D-TMR cells to HER3-DXd was similar to that of the respective parental cells.

**Fig. 2:**
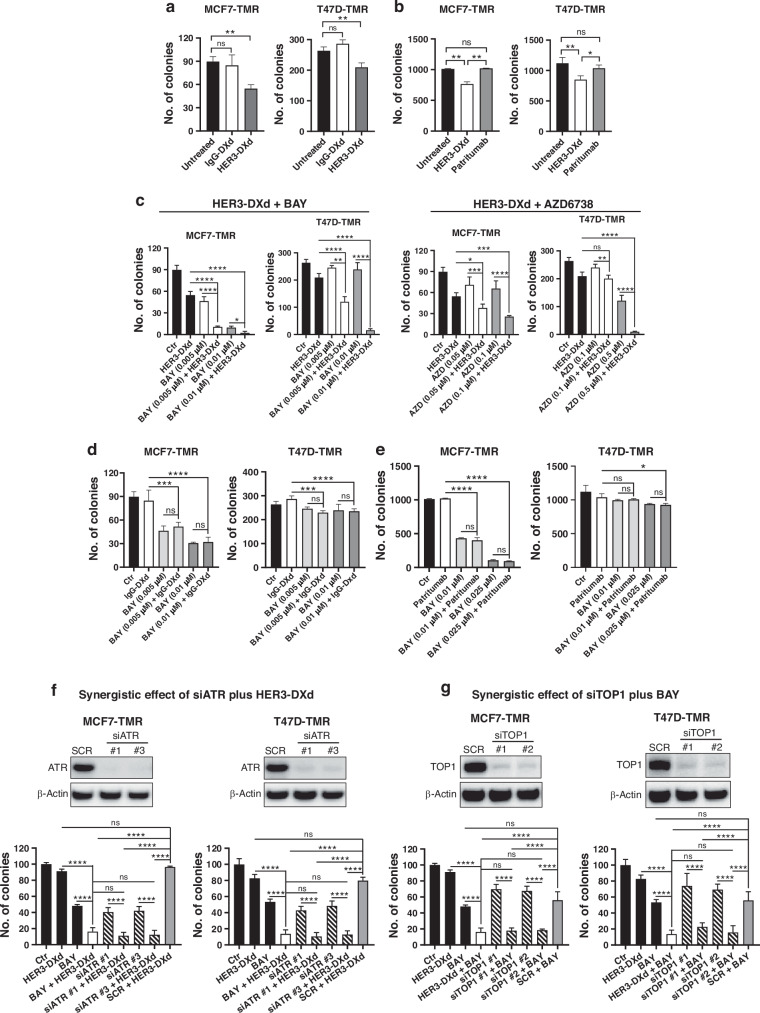
HER3-DXd inhibits MCF7-TMR and T47D-TMR cell growth through DXd, and targeting ATR enhances this effect. **a** Effects of HER3-DXd (5 nM) and IgG-DXd (5 nM) on colony formation in HER3+/HR+ MCF7-TMR and T47D-TMR cells as determined by clonogenic assay. **b** Effects of HER3-DXd (10 nM) and patritumab (10 nM) on anchorage-independent growth in MCF7-TMR and T47D-TMR cells as determined by soft agar assay. **c** Synergistic effects of HER3-DXd (10 nM) and ATR inhibitors (AZD6738 [AZD] and BAY 1895344 [BAY]) in MCF7-TMR and T47D-TMR cells as determined by clonogenic assay. Ctr, control (DMSO). Effects of (**d**) BAY plus IgG-DXd (5 nM) or (**e**) BAY plus patritumab (10 nM) on colony formation in MCF7-TMR and T47D-TMR cells as determined by clonogenic and soft agar assays, respectively. **f**, **g** HER3-DXd and BAY synergise by specifically targeting ATR and TOP1. **f** Knockdown of ATR using siRNA synergistically enhances the inhibiting effect of HER3-DXd (10 nM) on colony formation in HER3+/HR+ MCF7-TMR and T47D-TMR cells as determined by clonogenic assay. **g** Knockdown of TOP1 using siRNA synergistically enhances the inhibiting effect of BAY (0.01 µM) on colony formation in HER3+/HR+ MCF7-TMR and T47D-TMR cells as determined by clonogenic assay. Ctr, control (DMSO); SCR, scrambled control siRNA. Bars show the mean ± SD. ns: not significant, *P < 0.05, **P < 0.01, ***P < 0.001, ****P < 0.0001. Statistical significance was analysed by two-tailed unpaired Student’s *t*-tests. In **a**–**g**, the data are representative of triplicates from one of three independent experiments.

To verify whether the growth-inhibiting activity of HER3-DXd depended on its binding to HER3, we compared its growth-inhibiting activity with that of the IgG-DXd control. While HER3-DXd reduced anchorage-independent growth by 39.03% in MCF7-TMR and 14.09% in T47D-TMR cells, IgG-DXd had no effects (Fig. 2a), as determined by soft agar colony formation assay. Similar results were obtained with MCF7 and T47D cells (Fig. S2a). Furthermore, neither HER3-DXd nor IgG-DXd had effects on anchorage-independent growth in HER3-negative MDA-MB-231 and BCX-010 cells (Fig. S2b). These results suggest that the growth-inhibiting activity of HER3-DXd depends on its binding to HER3. To further verify whether HER3-DXd inhibits cell growth via suppressing HER3 signalling, we compared its growth-inhibiting activity with that of patritumab, which can block ligand-induced HER3 activation. Patritumab did not inhibit the growth of MCF7-TMR, T47D-TMR, MCF7, or T47D cells (Fig. 2b, S2c), as determined by clonogenic assay. This result suggests that HER3-DXd inhibits HER3+/HR+ cell growth primarily through the activity of the payload DXd.

### Targeting ATR enhanced the growth-inhibiting activity of HER3-DXd

To identify genes whose inhibition might enhance the growth-inhibiting activity of HER3-DXd, we performed high-throughput RNAi screening using the Ambio *Silencer* Select Human Genome siRNA Library V4 and MCF7 cells. On the basis of the fraction affected and a cutoff of a 0.15 sensitivity index score, we identified seven top genes, *ATR, ROCK2, RAB7A, CD247, UPK3A, SLC29A1/ENT1*, and *WNT7A* (Table S1 and S3), whose inhibition significantly enhanced the growth-inhibiting activity of HER3-DXd against MCF7 cells.

We further performed the soft agar colony formation assay to verify the synergistic effects of HER3-DXd with inhibition of the identified potential targets for which small molecular inhibitors are available, including ATR (BAY 1895344 and AZD6738; Fig. S4a), ROCK2 (azaindole 1; Fig. S4b), RAB7A (CID1067700; Fig. S4c), and SLC29A1/ENT1 (NBMPR; Fig. S4d). Among the tested combinations, HER3-DXd plus BAY 1895344 or AZD6738 exhibited the strongest synergistic effects. The synergy was further confirmed by clonogenic assay (Fig. 2c). In MCF7-TMR cells, HER3-DXd plus BAY 1895344 significantly reduced colony formation by 84.22-98.72% vs. HER3-DXd and by 77.62-90.5% vs BAY 1895344. In T47D-TMR cells, the combination significantly reduced colony formation by 36.46-99.19% vs. HER3-DXd and by 38.9-99.15% vs. BAY 1895344. HER3-DXd plus AZD6738 exhibited a similar pattern but had a lesser synergistic effect (Fig. 2c, S4a). Furthermore, synergistic effects were observed in MCF7 and T47D parental cells with HER3-DXd plus BAY 1895344 or AZD6738 (Fig. S5a). These results demonstrate that targeting ATR enhances the growth-inhibiting activity of HER3-DXd in HER3+/HR+ cells. Because HER3-DXd plus BAY 1895344 had a greater synergistic effect than HER3-DXd plus AZD6738 did, BAY 1895344 was chosen as the ATR inhibitor for the subsequent studies.

To verify whether the synergistic effect of HER3-DXd plus BAY 1895344 depends on HER-DXd’s binding to HER3, we treated cells with the IgG-DXd control plus BAY 1895344. The combination had no synergistic effect on the growth of MCF7-TMR, T47D-TMR, MCF7, or T47D cells (Figs. 2d, S5b). Additionally, the combination of HER3-DXd plus BAY 1895344 had no synergistic effects in HER3-negative MDA-MB-231 and BCX-010 cells (Fig. S5c). These results suggest that the synergy of HER3-DXd plus BAY 1895344 was HER3 dependent. To further verify whether HER3-DXd and BAY 1895344 synergistically inhibited the growth of HER3+/HR+ cells through suppression of HER3 signalling, we treated the cells with patritumab plus BAY 1895344. No synergy was observed (Figs. 2e, S5d). Taken together, these results demonstrate that the synergy of HER3-DXd plus BAY 1895344 is dependent on the binding of HER3-DXd to HER3 and is independent of HER3 signalling.

### HER3-DXd and BAY 1895344 synergised by specifically targeting TOP1 and ATR

To verify whether HER3-DXd and BAY 1895344 synergistically inhibited the growth of HER3+/HR+ cells by specifically targeting ATR and TOP1, we silenced the expression of ATR or TOP1 using siRNA and then treated the cells with HER3-DXd and BAY 1895344, respectively. Silencing ATR enhanced the growth-inhibiting activity of HER3-DXd by 78.88-80.13% in MCF7-TMR cells and by 69.87-72.19% in T47D-TMR cells (Fig. 2f). The synergistic effect of siATR plus HER3-DXd was similar to that of BAY 1895344 plus HER3-DXd. Similar results were obtained in parental MCF7 and T47D cells (Fig. S5e). Furthermore, silencing TOP1 enhanced the growth-inhibiting activity of BAY 1895344 by 29.73-30.31% in MCF7-TMR cells and by 30.64-37.68% in T47D-TMR cells (Fig. 2g). The synergistic effect of siTOP1 plus BAY 1895344 was similar to that of HER3-DXd plus BAY 1895344. Similar results were obtained in parental MCF7 and T47D cells (Fig. S5f). In contrast, no synergistic effects were observed when these cells were treated with scrambled control siRNA plus HER3-DXd (Figs. 2f, S5e) or BAY 1895344 (Figs. 2g, S5f). These results indicate that HER3-DXd synergises with BAY 1895344 by specifically targeting ATR and TOP1.

### BAY 1895344 enhanced the growth-inhibiting activity of HER3-DXd by increasing DNA damage

**Fig. 3:**
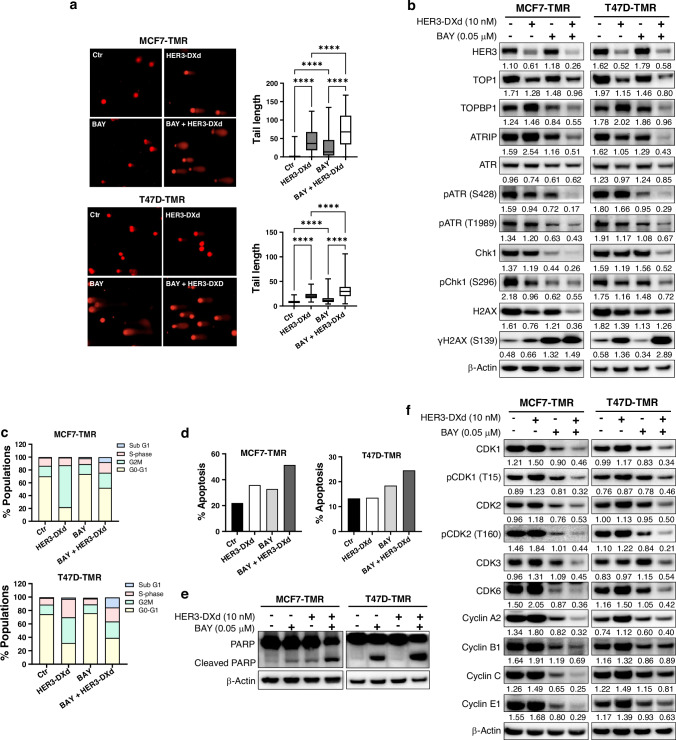
HER3-DXd and BAY 1895344 synergise by inducing DNA damage, cell cycle arrest, and apoptosis. **a** Comet formation (left) and quantification of comets formed (right) in HER3+/HR+ MCF7-TMR and T47D-TMR cells following treatment with DMSO (control; Ctr), HER3-DXd (10 nM), BAY 1895344 (BAY; 0.01 µM), or HER3-DXd plus BAY for 72 h. Bars show the mean ± SD. ns: not significant, ****P < 0.0001. Statistical significance was analysed by two-tailed unpaired Student’s *t*-tests. **b** Expression of proteins involved in DDR following treatment with the Ctr, HER3-DXd, BAY, or HER3-DXd plus BAY for 48 h as analysed by Western blotting. **c** Effects on cell cycle progression following treatment with the Ctr, HER3-DXd (10 nM), BAY (0.01 µM), or HER3-DXd plus BAY for 72 h as analysed by flow cytometry. **d** and **e** Apoptosis induction as analysed by **d** flow cytometry and **e** Western blotting following treatment with the Ctr, HER3-DXd (10 nM), BAY (0.05 µM), or HER3-DXd plus BAY for 72 h. **f** Expression of proteins regulating cell cycle progression following treatment with the Ctr, HER3-DXd, BAY, or HER3-DXd plus BAY for 48 h as analysed by Western blotting. In (**b**) and (**f**), β-Actin was used as a loading control. Band intensity of proteins is normalised to that of β-Actin. In (**a,** **c**, and **d**), the data are representative of triplicates from one of three independent experiments. In (**b,** **e**, and **f**), the data are representative of triplicates from one of two independent experiments.

Given that ATR plays a key role in DDR, we examined whether BAY 1895344 synergised with HER3-DXd by increasing DNA damage using the comet assay. Compared with the DMSO control, treatment with HER3-DXd or BAY 1895344 significantly increased comet formation (an indicator of DNA damage) in both MCF7-TMR cells (86.36% by HER3-DXd; 77.96% by BAY 1895344; Fig. 3a) and T47D-TMR cells (54.39% by HER3-DXd; 38.97% by BAY 1895344; Fig. 3a). Furthermore, compared with HER3-DXd or BAY 1895344 monotherapy, the combination induced more comet formation in both MCF7-TMR cells (40.59% vs HER3-DXd; 63.24% vs BAY 1895344; Fig. 3a) and T47D-TMR cells (32.3% vs HER3-DXd; 49.4% vs BAY 1895344; Fig. 3a). Similar results were seen in parental MCF7 and T47D cells (Fig. S6a). These results suggest that the synergistic growth inhibition is a result of increased DNA damage, which was further confirmed by increased expression of γH2AX at Ser139 (Figs. 3b, S6c), an indicator of DNA damage [27].

ATR is a key player in responses to DNA damage and replication stress. Therefore, we examined the treatments’ effects on expression of the target proteins (HER3, ATR, and TOP1) as well as proteins related to DNA damage (H2AX and γH2AX) and replication stress (TOPBP1, ATRIP, and Chk1) by Western blotting. Expression levels of these proteins were comparable between MCF-7-TMR and parental MCF-7 cells, as well as between T47D-TMR and parental T47D cells (Fig. S6b). As shown in Fig. 3b and S6c, treatment with HER3-DXd alone markedly reduced HER3 expression, likely reflecting lysosomal degradation of HER3 following HER3-DXd binding and internalisation. Compared with HER3-DXd or BAY 1895344 monotherapy, the combination dramatically reduced TOP1 and Chk1 expression, ATR phosphorylation at Ser428 and Thr1989, and Chk1 phosphorylation at Ser296. ATR/Chk1 signalling halts cell cycle progression and promotes DDR upon DNA damage and replication stress [16, 17]. We also examined the effects of the treatments on the expression of proteins required for ATR activation, including RPA, TOPBP1, ATRIP, and ETAA1 [28]. Compared with HER3-DXd or BAY 1895344 monotherapy, the combination dramatically reduced expression of TOPBP1 and ATRIP (Figs. 3b, S6c) and had no effects on expression and phosphorylation of RPA and ETAA1 (data not shown). As TOPBP1 and ATRIP are essential for ATR activation and Chk1 is a downstream effector of ATR, these results suggest that HER3-DXd and BAY 1895344 synergise by suppressing ATR/Chk1 signalling activation, leading to DNA damage accumulation and enhanced cell death.

### BAY 1895344 enhanced the growth-inhibiting activity of HER3-DXd by arresting cell cycle progression and inducing apoptotic cell death

To further characterise the mechanism of action of HER3-DXd plus BAY 1895344, we examined the treatments’ impact on cell cycle progression because ATR is a key regulator of DDR in response to replication stress [16]. Compared with the DMSO control, HER3-DXd increased the proportion of cells in G2M phase by 74.99% in MCF7-MTR cells and by 62.46% in T47D-MTR cells, whereas BAY 1895344 increased the proportion of cells in sub-G1 phase by 61.91% in MCF7-MTR cells and by 20.64% in T47D-MTR cells (Fig. 3c). Compared with HER3-DXd alone, the combination reduced the proportion of cells in G2M phase by 64.06% in MCF7-MTR cells and by 35.84% in T47D-MTR cells, indicating that ATR inhibition released the cells from HER3-DXd–induced G2/M arrest. Furthermore, compared with HER3-DXd or BAY 1895344 monotherapy, the combination increased the proportion of cells in the sub-G1 phase in both MCF7-TMR cells (by 91.39% vs HER3-DXd; 75.71% vs BAY 1895344) and T47D-TMR cells (by 86.52% vs HER3-DXd; 91.55% vs BAY 1895344). Similar effects were observed in parental MCF7 and T47D cells (Fig. S6d). The increase in sub-G1 phase following the combination treatment indicates the induction of apoptosis, which was further confirmed by Annexin V-PE and 7-AAD staining (Figs. 3d, S6e) and increased expression of cleaved PARP (Figs. 3e, S6f). The combination increased the apoptosis in both MCF7-TMR cells (by 30.21% vs HER3-DXd; 36.2% vs BAY 1895344) and T47D-TMR cells (by 44.85% vs HER3-DXd; 24.99% vs BAY 1895344; Fig. 3d). Similar effects were observed in parental MCF7 and T47D cells (Fig. S6e). These results demonstrate that BAY 1895344 enhanced the growth-inhibiting activity of HER3-DXd by releasing cells from HER3-DXd–induced G2/M arrest, which in turn led to DNA damage accumulation and increased apoptotic cell death.

Given that CDKs and cyclins play critical roles in cell cycle progression, we examined the treatments’ effects on the expression of CDKs and cyclins by Western blotting. As shown in Figs. 3f and S6g, compared with HER3-DXd or BAY 1895344 monotherapy, the combination dramatically reduced expression of CDK1, phospho-CDK1 (pCDK1) at Tyr15, CDK2, phospho-CDK2 (pCDK2) at Thr160, CDK3, CDK6, cyclin A2, and cyclin E1 in MCF7-TMR, T47D-TMR, MCF7, and T47D cells. The combination also reduced expression of cyclin B1 and cyclin C in MCF7 and MCF7-TMR cells but not in T47D and T47D-TMR cells. No effects were seen on CDK4, CDK7, and cyclin D1 (data not shown). These results suggest that HER3-DXd and BAY 1895344 synergise by reducing the expression of cell cycle regulatory proteins, leading to cell cycle arrest.

### BAY 1895344 enhanced the antitumor efficacy of HER3-DXd in TMR mouse models

**Fig. 4:**
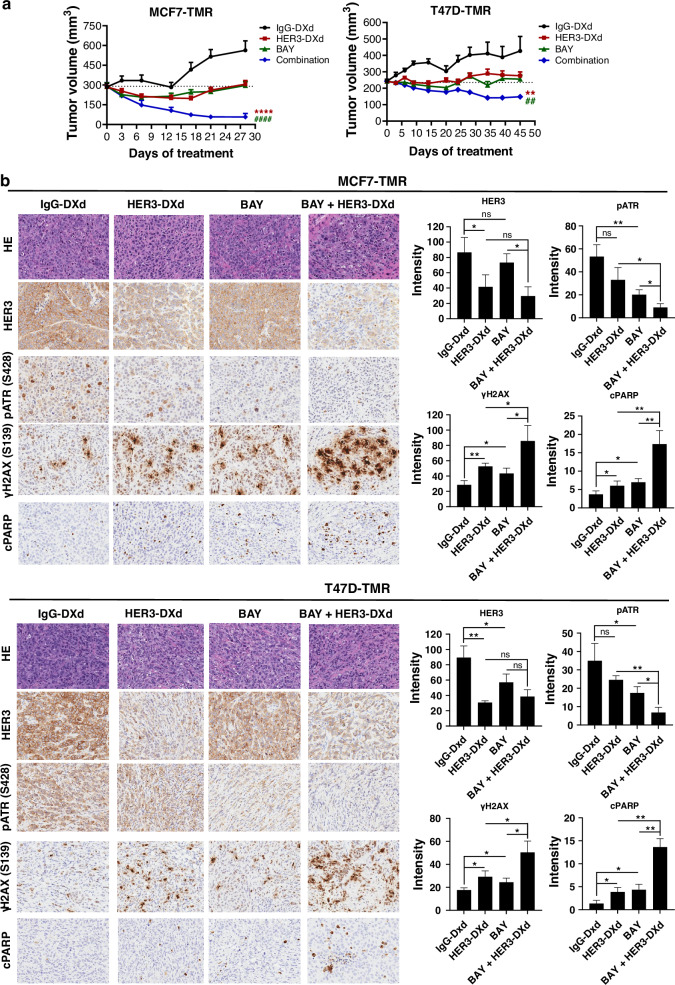
HER3-DXd and BAY 1895344 synergistically inhibit tumour growth in MCF7-TMR and T47D-TMR xenograft models. Effects of IgG-DXd (1 mg/kg, weekly, intravenously), HER3-DXd (1 mg/kg, weekly, intravenously), BAY 1895344 (BAY; 7.5 mg/kg, twice a day, 3 days/week, orally), and HER3-DXd plus BAY on (**a**) tumour growth and (**b**) target protein expression in MCF7-TMR and T47D-TMR xenograft mouse models. Images were captured at 20× magnification. In **a**, Bars show the mean ± SD. **P < 0.01 and ****P < 0.0001 for the difference in tumour volume between the mice treated with HER3-DXd and the mice treated with HER3-DXd plus BAY. **##** *P* < 0.01 and #### *P* < 0.0001 for the difference in tumour volume between the mice treated with BAY and the mice treated with HER3-DXd and BAY. In (**b**), bars show the mean ± SD. ns: not significant, *P < 0.05, **P < 0.01. Differences in tumour volume between treatment groups were analysed using two-way ANOVA. Post hoc comparisons were Bonferroni-adjusted, with statistical significance set at *P* ≤ 0.05.

Building on our in vitro results, we next tested whether BAY 1895344 enhances the antitumor efficacy of HER3-DXd in vivo. We treated NSG mice bearing MCF7-TMR or T47D-TMR tumours with IgG-DXd (1 mg/kg, weekly, intravenously), HER3-DXd (1 mg/kg, weekly, intravenously), BAY 1895344 (7.5 mg/kg, 3 days/week, orally), or HER3-DXd plus BAY 1895344. Compared with IgG-DXd, HER3-DXd alone and BAY 1895344 alone significantly suppressed tumour growth in mice bearing MCF7-TMR or T47D-TMR tumours (Fig. 4a). Furthermore, compared with HER3-DXd or BAY 1895344 monotherapy, the combination had a greater tumour-inhibiting activity. No significant changes were observed in mouse body weights following the combination treatment in the MCF7-TMR model, indicating that mice tolerated the combination treatment well (Fig. S7). In contrast, a modest but consistent 10–15% decrease in body weight was noted in the T47D-TMR model across all treatment arms, including the IgG-DXd control. This pattern suggests that the decrease in body weight was not treatment-related but likely reflected baseline variability in the health condition or metabolic status of the mouse batch used for this model. Overall, these results suggest that targeting ATR could improve the antitumor efficacy of HER3-DXd in HER3+/HR+ TMR BC.

We next examined the treatments’ effects on the expression of target proteins HER3 and ATR in tumours by immunohistochemistry staining. As shown in Fig. 4b, expression levels of HER3 were significantly lower in tumours treated with HER3-DXd than in tumours treated with IgG-DXd in both MFC7-TMR and T47D-TMR models. No difference in HER3 expression was found between tumours treated with HER3-DXd and those treated with HER3-DXd plus BAY 1895344 in either model. Reduced HER3 expression likely reflects lysosomal degradation of HER3 following HER3-DXd binding and internalisation, consistent with the Western blot results (Figs. 3b, S6c). Expression levels of pATR at Ser428 were lower in tumours treated with BAY 1895344 than in tumours treated with IgG-DXd in both models. A reduction in expression of pATR at Ser428 was also observed in HER3-DXd-treated tumours compared to IgG-DXd-treated tumours in both models, but this did not reach statistical significance. Compared with HER3-DXd or BAY 1895344 monotherapy, the combination significantly reduced expression of pATR at S428 in both models.

Building on our in vitro observation that the combination treatment synergistically suppressed cell growth by inducing DNA damage (Figs. 3a, S6a) and subsequent apoptosis (Figs. 3d, e, S6e, S6f), we assessed the treatments’ effects on the expression of γH2AX (Ser139) and cPARP. Expression levels of γH2AX (Ser139) and cPARP were higher in tumours treated with HER3-DXd or BAY 1895344 than in tumours treated with IgG-DXd in both models (Fig. 4b). Expression levels of γH2AX (Ser139) and cPARP were significantly higher in tumours treated with HER3-DXd plus BAY 1895344 than in tumours treated with the monotherapies in both models (Fig. 4b). Our data collectively indicate that BAY 1895344 enhances the antitumor efficacy of HER3-DXd by inducing DNA damage and subsequent apoptotic cell death.

### Prognostic impact of target genes involved in the antitumor synergy of HER3-DXd and BAY 1895344 in HR+ and overall BC

**Fig. 5:**
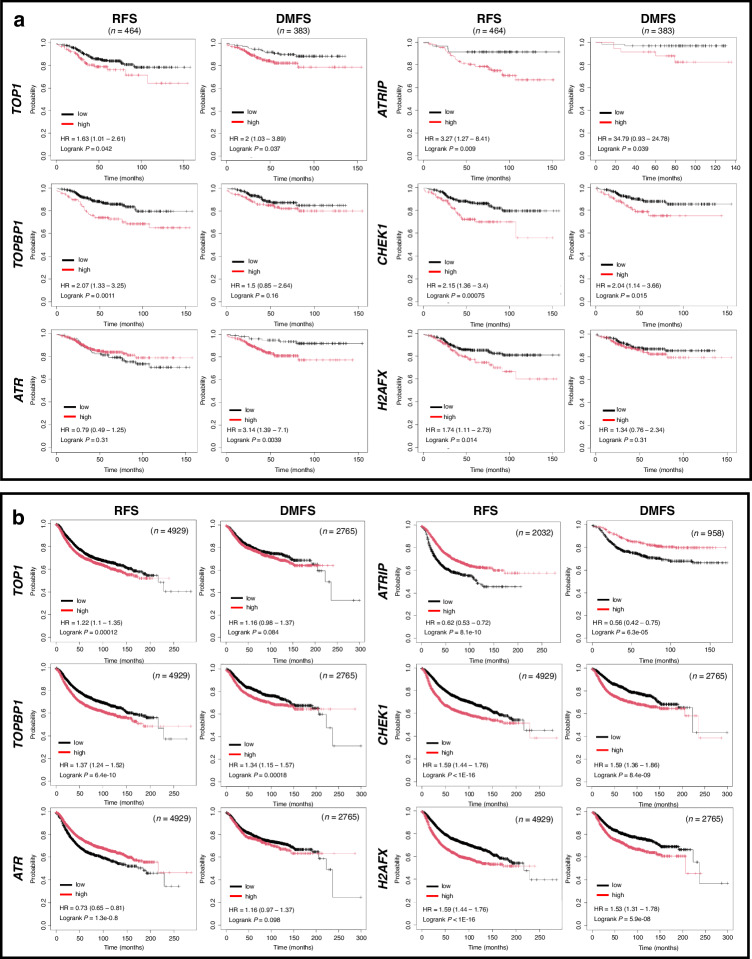

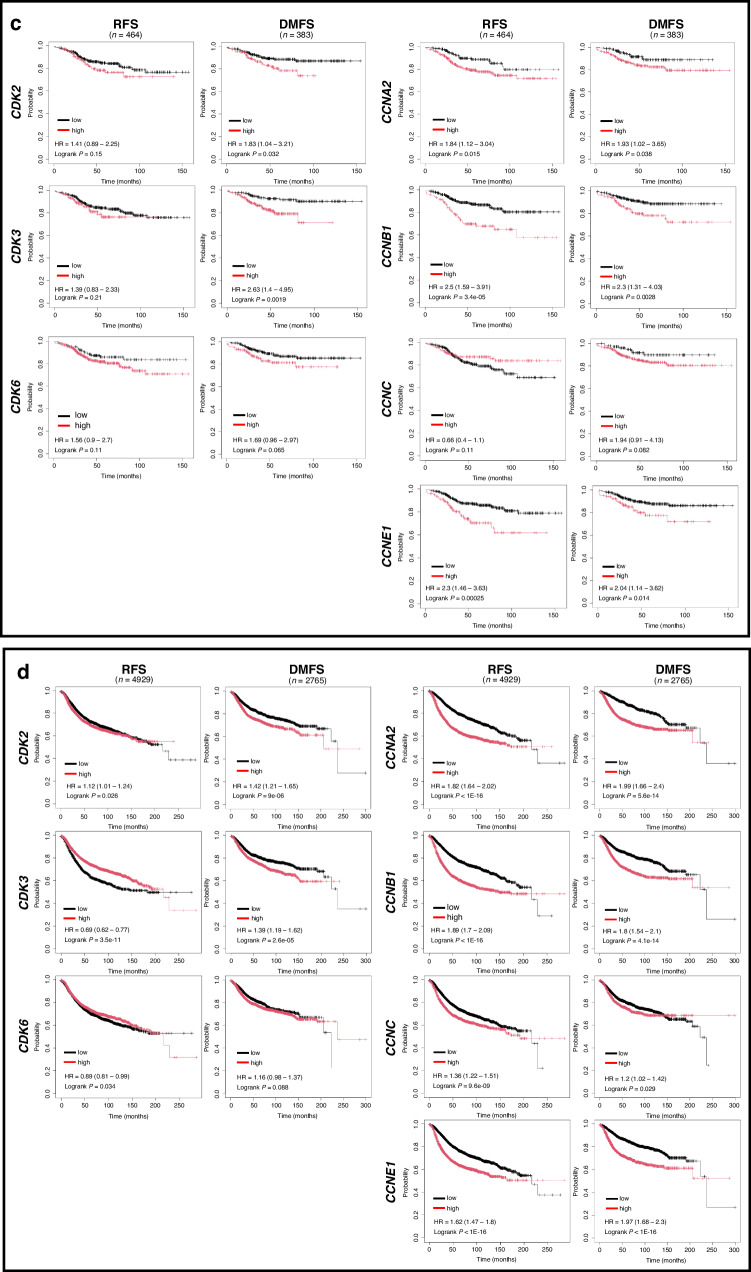
Prognostic impact of target gene expression in HR+ and overall BC. The prognostic impact of (**a**, **b**) genes involved in DNA damage and repair and (**c**, **d**) genes regulating cell cycle progression was analysed using TCGA RNA sequence data from (**a**, **c**) patients with HR+ BC treated with endocrine therapy alone or in combination with chemotherapy, and (**b**, **d**) all BC patients irrespective of subtype. Analyses were performed using UALCAN and Kaplan–Meier Plotter. RFS, recurrence-free survival, DMFS distant metastasis–free survival.

Given that HER3-DXd and BAY 1895344 synergise by suppressing expression of proteins involved in DDR and cell cycle progression, we assessed the prognostic impact of mRNA levels of their corresponding genes, including *TOP1*, *TOPBP1*, *ATR*, *ATRIP*, *CHEK1*, and *H2AFX*, in HR+ BC treated with systematic endocrine therapy, either alone or in combination with chemotherapy, using the TCGA dataset (Fig. 5a). Kaplan–Meier Plotter analysis revealed that high mRNA levels of *TOP1*, *TOPBP1*, *ATRIP*, *CHEK1*, and *H2AFX* correlated with worse RFS, while no correlation was found for *ATR*. Further, high mRNA levels of *TOP1*, *ATR*, and *CHEK1* correlated with worse DMFS, while no correlations were found for *TOPBP1*, *ATRIP*, and *H2AFX*. Among these genes, *TOPBP1* and *CHEK1* showed the strongest associations with poor RFS in endocrine therapy–treated HR+ BC. We next examined whether these gene expression–survival correlations were maintained in overall BC using the TCGA dataset (Fig. 5b). High mRNA levels of *TOP1*, *TOPBP1*, *CHEK1*, and *H2AFX* strongly correlated with worse RFS, whereas *ATR* and *ATRIP* showed a strong correlation with better RFS. Furthermore, high mRNA levels of *TOPBP1*, *CHEK1*, and *H2AFX* strongly correlated with worse DMFS, whereas *ATRIP* showed a strong correlation with better DMFS. No correlations were found between *TOP1* or *ATR* and DMFS.

We also assessed the prognostic impact of mRNA levels of genes involved in cell cycle progression, including *CDK2*, *CDK3*, *CDK6*, *CCNA2*, *CCNB1*, *CCNC*, and *CCNE1*, in HR+ BC using the same dataset used for DDR analysis (Fig. 5c). High mRNA levels of *CCNA2*, *CCNB1*, and *CCNE1* correlated with worse RFS, while those of *CDK2*, *CDK3*, *CCNA2*, *CCNB1*, and *CCNE1* correlated with worse DMFS. No correlations were found between *CDK2*, *CDK3*, *CDK6*, or *CCNC* and RFS or between *CDK6* or *CCNC* and DMFS. Among these genes, *CCNB1* showed the strongest correlation with both poor RFS and poor DMFS in endocrine therapy–treated HR+ BC. We also evaluated their clinical relevance in overall BC using the same dataset used for DDR analysis (Fig. 5d). High mRNA levels of *CDK2*, *CCNA2*, *CCNB1*, *CCNC*, and *CCNE1* correlated with worse RFS and DMFS, while high *CDK3* mRNA levels correlated with better RFS and worse DMFS in overall BC. High *CDK6* mRNA levels correlated with better RFS and had no correlation with DMFS.

In summary, our analysis demonstrates that across both endocrine therapy–treated HR+ BC and overall BC, several DDR– and cell cycle–related genes were consistently associated with unfavourable outcomes. Specifically, *CHEK1*, *CCNA2*, *CCNB1*, and *CCNE1* expression correlated with worse RFS and DMFS, while *TOP1*, *TOPBP1*, and *H2AFX* correlated with worse RFS, and *CDK2* and *CDK3* correlated with worse DMFS. Interestingly, *ATR* expression showed a trend toward better RFS but worse DMFS in HR+ BC and a correlation with better RFS and a trend toward worse DMFS in overall BC. *CDK6* displayed a trend toward worse RFS and DMFS in HR+ BC and a correlation with worse RFS and DMFS in overall BC. Notably, discordant associations were observed for *ATRIP* and *CCNC*. *ATRIP* correlated with worse RFS and trended toward worse DMFS in HR+ BC but correlated with better RFS and DMFS in overall BC. *CCNC* trended toward better RFS but worse DMFS in HR+ BC, whereas it correlated with worse RFS and DMFS in overall BC. These findings suggest that the prognostic significance of individual DDR– and cell cycle–related genes may differ depending on BC subtypes or treatment context.

## Discussion

**Fig. 6:**
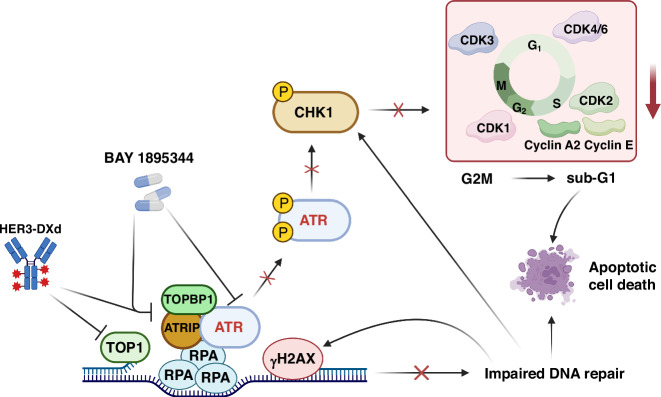
Diagram showing the molecular mechanism underlying the synergy of HER3-DXd and BAY 1895344. HER3-DXd and BAY 1895344 (BAY) synergistically inhibit expression of TOPBP1 and ATRIP, which are essential for ATR activation, leading to suppression of ATR phosphorylation at both Ser428 and T1989 and subsequent suppression of Chk1 expression and phosphorylation at S296. Reduced expression of TOPBP1 and ATRIP and suppressed ATR activation prevent their binding to DNA breaks, impairing DDR and leading to accumulation of DNA damage, which increases γH2AX (S139) expression. Reduced Chk1 expression and activation further result in a reduction in expression of CDK1, pCDK1, CDK2, CDK3, pCDK2, CDK4, CDK6, cyclin A2, and cyclin E, leading to cell cycle arrest at sub-G1. Impaired DDR and cell cycle arrest at sub-G1 induce apoptosis.

Acquired resistance to endocrine therapy is a major challenge in HR+ BC, underscoring the need for more effective treatment strategies. In this study, we demonstrated that HER3-DXd effectively inhibited the growth of TMR HER3+/HR+ BC cells both in vitro and in vivo. Combining HER3-DXd with the ATR inhibitor BAY 1895344 further enhanced antitumor efficacy by impairing DDR through downregulation of DDR-related proteins (Fig. 6). The combination also induced sub-G1 cell cycle arrest via inhibition of both ATR/Chk1/cyclin A2/CDK2 and ATR/Chk1/cyclin E1/CDK2 signalling, leading to apoptotic cell death (Fig. 6). These findings support the potential of combining HER3-DXd with ATR inhibitors as a promising strategy for HER3+/HR+ BC, including endocrine therapy–resistant disease.

DDR pathways are frequently altered and activated in endocrine therapy–resistant ER+ BC, driving tumour progression and treatment evasion. Resistant tumour cells often acquire an enhanced DNA repair capacity, which enables survival under DNA damage and replicative stress. This is frequently associated with alterations in DDR genes and elevated DDR protein expression. The changes in DDR capacity and DDR gene and protein expression collectively facilitate resistance to endocrine therapy [29]; crosstalk between ER and DDR signalling further contributes to this phenotype [30]. Given DDR’s critical role in endocrine resistance, targeting DDR represents a promising strategy to overcome this therapeutic challenge. For instance, PARP inhibitors, designed to disrupt DNA repair [31], have demonstrated efficacy in DDR-altered ER+ BC [32, 33] and re-sensitised endocrine therapy–resistant ER+ BC cells to oestrogen therapy [30]. Moreover, strategies combining PARP inhibitors with ATR, WEE1, or DNA ligase inhibitors have exhibited efficacy in preclinical models [34]. Collectively, these findings highlight DDR activation and/or alterations as a hallmark of endocrine therapy–resistant ER+ BC and support the rationale for clinical development of DDR-targeted therapies, either alone or in combination with endocrine treatments.

Our rationale for combining ATR inhibition with HER3-DXd was to exploit the adaptive DDR, as ATR inhibition impairs the repair of HER3-DXd–induced DNA damage and thereby enhances cytotoxicity. Mechanistically, BAY 1895344 reduced ATR phosphorylation at both Ser428 and Thr1989, and the combination further suppressed ATR and Chk1 phosphorylation, indicating effective inhibition of ATR/Chk1 signalling. This inhibition impaired DDR and abrogated HER3-DXd–induced G2/M arrest, leading to unrepaired DNA damage accumulation and apoptotic cell death. Our findings highlight the therapeutic potential of combining ADCs with DDR inhibitors. Several such combinations are under clinical evaluation, including sacituzumab govitecan with the PARP inhibitor talazoparib for metastatic TNBC (NCT04039230) [35], T-DXd with olaparib for HER2+ cancers (NCT04585958, module 2) [36], and trastuzumab duocarmazine (SYD985) with niraparib for HER2+ advanced BC or metastatic BC progressed on standard therapy (NCT04235101). Some of these combinations have demonstrated feasibility and preliminary efficacy (NCT04039230; NCT04585958, module 2) [35, 36]. HER3-DXd is also being evaluated in combination with olaparib in HER2+ and HER2-low advanced BC that progressed on T-DXd in the ICARUS-BREAST02 study (NCT06298084, module 1). In addition to PARP inhibitors, other DDR inhibitors are being tested in clinical trials with ADCs. For example, a Phase I/II clinical trial is currently evaluating the ATR inhibitor berzosertib combined with sacituzumab govitecan in cancers resistant to PARP inhibitors (NCT04826341). In our study, ATR inhibitors enhanced the antitumor efficacy of ADCs with payloads that induce DNA damage. Similarly, combinations of T-DXd with BAY 1895344, or with both BAY 1895344 and olaparib, have shown synergistic effects in HER2+ preclinical models [37, 38]. These clinical and preclinical studies support the concept of developing synthetic lethality by combining ADCs with DDR inhibitors as a promising strategy for BC. However, overlapping and cumulative toxicities remain a challenge [39], underscoring the importance of close monitoring and proactive management to maintain treatment tolerability and optimise outcomes.

BAY 1895344 has been evaluated in early-phase clinical trials as monotherapy and in combination regimens for advanced solid tumours and lymphomas (NCT03188965). As a single agent, it has shown modest antitumor efficacy, with partial responses and disease stabilisation observed in tumours harbouring replication stress or DDR alterations, including HR+/HER2-negative BC [40, 41]. The most common adverse events are haematologic, e.g., neutropenia, anaemia, thrombocytopenia, and leukopenia, along with mucositis, gastrointestinal toxicities, and fatigue (NCT04514497) [41–43]. These adverse events are generally reversible with treatment interruption or dose modification [40]. In combination settings, BAY 1895344 has been tested with chemotherapy, PARP inhibitors, immunotherapy, and radiotherapy. For instance, in a Phase I trial with TOP1 inhibitors (irinotecan or topotecan) for advanced solid tumours, partial response (12.5%) and disease stabilisation (25%) were reported, though dose escalation was limited by myelotoxicity (NCT04514497) [43, 44]. Ongoing trials are also evaluating BAY 1895344 with niraparib (NCT04267939), gemcitabine (NCT04616534), pembrolizumab (NCT04095273), and pembrolizumab plus radiotherapy (NCT04576091) [45]. These studies have demonstrated promising efficacy but highlight dose-limiting haematologic toxicity as a key limitation of ATR inhibition. HER3-DXd exhibits promising antitumor activity but with a notable toxicity profile, e.g., gastrointestinal (nausea, vomiting), haematologic (neutropenia and thrombocytopenia), and fatigue-related adverse events, as well as the ADC-class risk of interstitial lung disease/pneumonitis due to its DXd payload [12, 46]. Given these overlapping toxicities, cumulative adverse effects are anticipated when BAY-1895344 is combined with HER3-DXd. While the mechanistic rationale supports synergy between ATR inhibition and the DNA-damaging DXd payload, cautious clinical development is warranted. Early-phase studies should employ stepwise dose escalation, sequential versus concurrent dosing evaluation, and close haematologic and pulmonary monitoring to balance efficacy with safety.

Given the frequent DDR activation and alterations and high HER3 expression in HR+ BC, particularly in endocrine therapy–resistant disease, combining BAY 1895344 with HER3-DXd may offer a promising strategy to enhance therapeutic efficacy and overcome endocrine resistance. Building on our preclinical findings, the combination may synergistically inhibit tumour growth by enhancing DNA damage through dual inhibition of TOP1 and ATR. HER3-DXd provides improved tumour-targeting specificity, while ATR inhibition amplifies the DNA-damaging effect, potentially overcoming endocrine resistance. This rationale is supported by early-phase clinical studies in which BAY 1895344 combined with TOP1 inhibitors demonstrated encouraging antitumor activity in advanced solid tumours (NCT04514497) [43]. Together, our findings support the translational potential of HER3-DXd plus BAY 1895344 as a therapeutic strategy for HER3+/HR+ BC, particularly in the endocrine therapy–resistant setting.

Our prognostic analysis of genes associated with DDR and cell cycle regulation revealed that high *CCNB1* mRNA levels exhibited the strongest association with worse RFS and DMFS in HR+ BC, consistent with previous studies linking elevated *CCNB1* expression to aggressive features and poor outcomes [47]. High *CCNA2* and *CCNE1* mRNA levels were also modestly associated with worse RFS and DMFS; while *CCNA2*’s prognostic role in ER+ BC is well established [48], the impact of *CCNE1* remains less defined. *CCNC*, though less studied, has recently emerged as a potential prognostic biomarker in BC [49], warranting further validation. Associations for other genes examined, including *ATR*, *ATRIP*, *TOP1*, *TOPBP1*, *CHEK1*, *H2AFX*, and *CDKs*, were modest and have not been extensively reported in HR+ BC. For instance, although *ATR* has been studied in other cancers [50], its prognostic role in HR+ BC remains to be fully defined. Notably, *ATRIP* mutations and elevated *CHEK1 and TOPBP1* expression have been linked to aggressive tumour features [51–53], but their correlations with outcomes in HR+ BC are less established. Although elevated *H2AFX* and *CDK3* mRNA levels and CDK2 protein expression have been correlated with poor outcomes in BC [54–57], their prognostic roles in HR+ BC are not well defined. Overall, our findings corroborate the known prognostic roles of *CCNA2* and *CCNB1* and highlight potential contributions of additional DDR and cell cycle–related genes, supporting the clinical relevance of targeting pathways such as ATR/Chk1 in this subtype.

In summary, our study demonstrates that combining HER3-DXd with ATR inhibition represents a promising strategy to overcome endocrine resistance in HER3+/HR+ BC by impairing DDR signalling, inducing cell cycle arrest, and promoting apoptosis. Prognostic analyses further support the clinical relevance of DDR- and cell cycle–related genes, reinforcing the rationale for this therapeutic strategy. Our findings highlight the potential of combining HER3-DXd with DDR inhibitors to enhance treatment efficacy and overcome endocrine therapy resistance in HR+/HER3+ BC and warrant further clinical evaluation with careful optimisation of dosing and toxicity management.

## Supplementary information

Supplementary Material

## Data Availability

The data that support the findings of this study are available from the corresponding author upon reasonable request.
