# Supplementary material for: ATR inhibition potentiates the antitumor efficacy of HER3-DXd in HER3-positive/HR-positive breast cancer by increasing DNA damage
Source: Br J Cancer. 2026 Apr 15;135(1):33–47. doi: 10.1038/s41416-026-03413-1 (PMC13270164; doi:10.1038/s41416-026-03413-1)
Supplement: Supplementary file 1 — Supplementary Material [file 41416_2026_3413_MOESM1_ESM.docx]

**Supplementary Information**

**Supplementary Materials and Methods**

**Determination of HER3 expression on cell surfaces**

Cells (5 × 10^5^ cells) were seeded in 60-mm plates and cultured for 48 h at 37 ^o^C. The cells were collected, stained with phycoerythrin-conjugated anti-HER3 antibody (#77956; Cell Signaling Technology, Danvers, MA, USA) for 15 min at 4 ^o^C, and then analyzed for HER3 expression on the cell surface by flow cytometry.

**Determination of HER3-DXd internalization into HER3+/HR+ BC cells**

The internalization of HER3-DXd into HER3+/HR+ BC cells was determined using the pHAb Amine Reactive Dye (# G9841; Promega Corporation, Madison, WI, USA) per the manufacturer’s instructions. HER3-DXd (100 µg) was mixed with the pHAb amine reactive dye (1.2 uL of 10 mg/mL) and incubated for 1 h at room temperature. After incubation, the unreacted dye was removed using a Zeba protein desalting column (#89882; Thermo Fisher Scientific). The resulting HER3-DXd-Dye conjugates (10 nM) were added into 96-well plates containing HER3+ or HER3-negative BC cells (20,000 cells/well), followed by 24-h incubation at 37 ^o^C. The internalized conjugates were measured at Ex 532 nm/Em 560 nm using a Victor X3 plate reader (PerkinElmer, Waltham, MA, USA).

**siRNA transfection**

Cells (5 × 10^5^) were transfected with scrambled control siRNA or siRNA that targets ATR or TOP1 (Sigma-Aldrich) at a final concentration of 5 µM using the Neon Transfection System (Life Technologies Corporation, Carlsbad, CA, USA).

**Western blot analysis**

Western blot analysis was performed as described previously (1). Cells (2 × 10^6^ cells) were seeded in 10-cm plates for overnight and treated the next day with HER3-DXd (10 nM), BAY 1895344 (0.05 µM), or HER3-DXd plus BAY 1895344 for 48 h at 37 °C. After treatment, total proteins were extracted using M-PER Mammalian Protein Extraction solution (#78501; ThermoFisher Scientific) containing phosphatase and protease inhibitor cocktails (#B15001; Bimake, Houston, TX, USA) and then analyzed by Western blotting.

**References**

1. Bartholomeusz C, Gonzalez-Angulo AM, Kazansky A, Krishnamurthy S, Liu P, Yuan LX, et al. PEA-15 inhibits tumorigenesis in an MDA-MB-468 triple-negative breast cancer xenograft model through increased cytoplasmic localization of activated extracellular signal-regulated kinase. Clin Cancer Res. 2010;16(6):1802-11.

**Supplementary Figures**

**Supplementary Fig. S1: HER3 expression on the surface of HR+ BC cells, HER3-DXd internalization into HER3+/HR+ BC cells, and growth-inhibiting activity of HER3-DXd against HER3+/HR+ BC cells.**

**
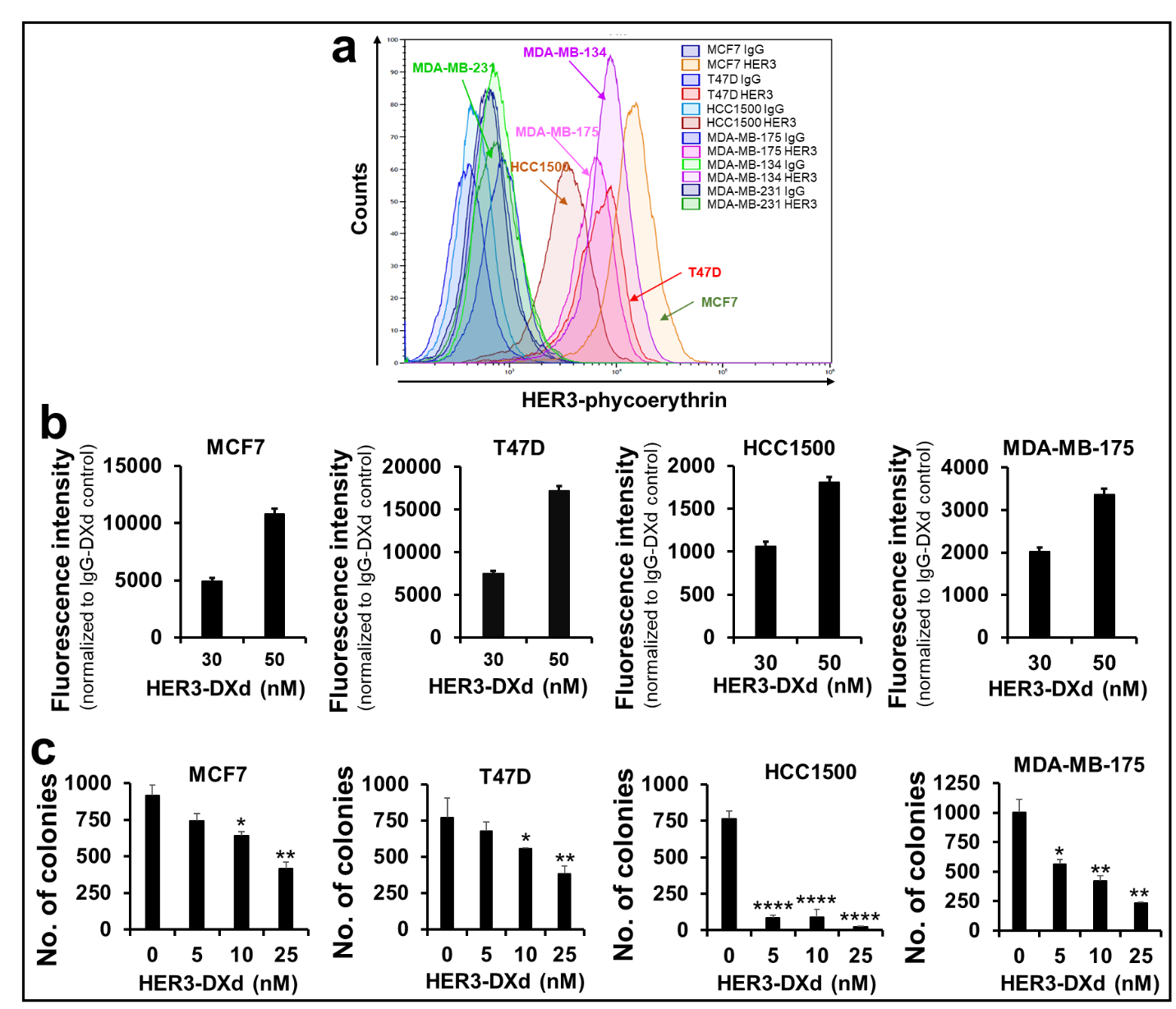
**

**a** HER3 expression on the surface of ER+ cells as determined by flow cytometry. **b** Internalization of HER3-DXd into HER3+/HR+ cells as measured using a pHAb Amine Reactive Dye. **c** HER3-DXd inhibits colony formation as determined by clonogenic assay in HER3+/HR+ cells. Bars show the mean ± SD. **P* < 0.05, ***P* < 0.01, *****P* < 0.0001, vs. the untreated control. In **a** and **b**, the data are representative of triplicates from one of two independent experiments. In **c**, the data are representative of triplicates from one of three independent experiments.

**Supplementary Fig. S2:** **Effects of HER3-DXd, IgG-DXd, and patritumab on the growth of BC cells.**


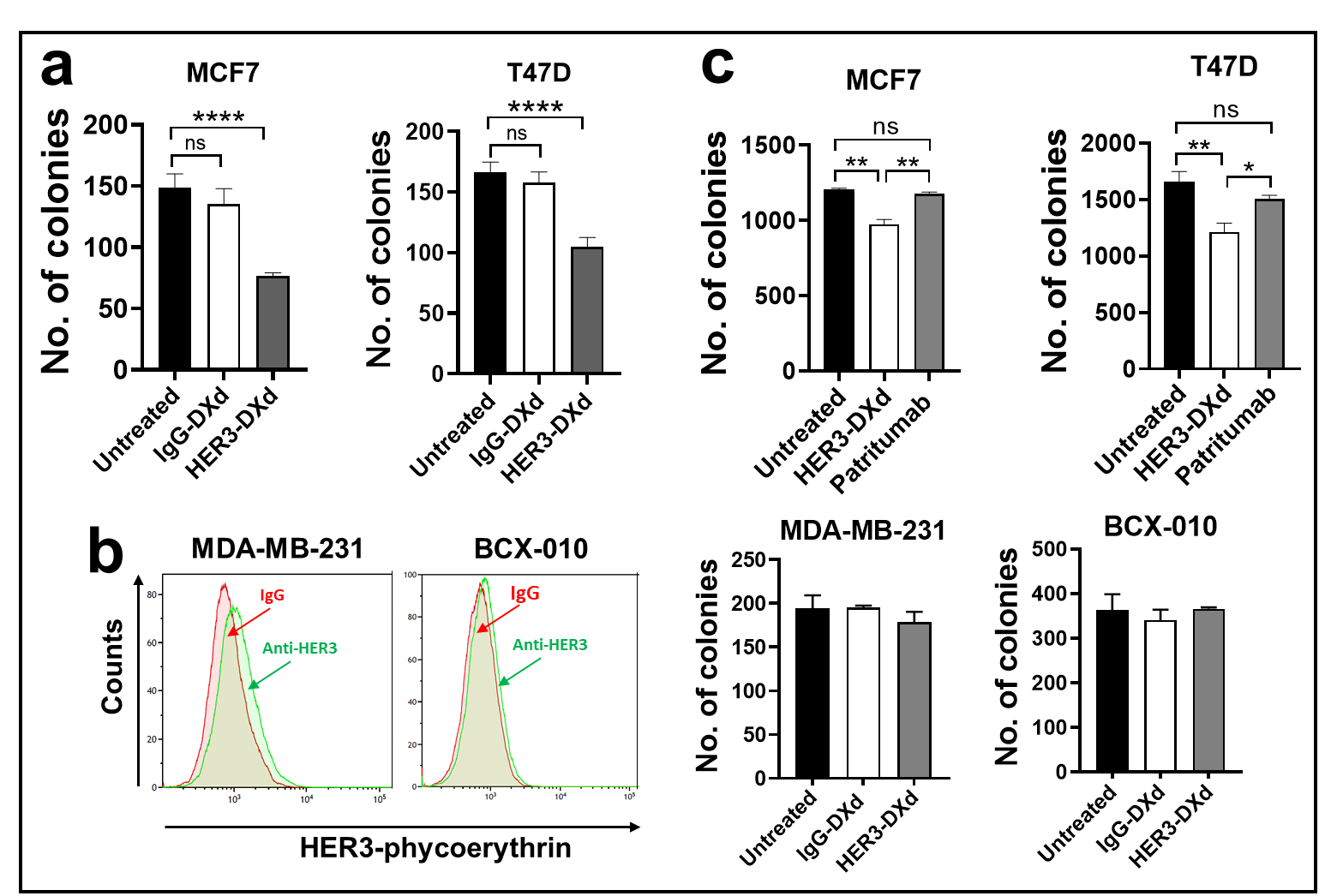


**a** and **b** Effects of HER3-DXd (5 nM) and IgG-DXd (5 nM) on anchorage-independent growth in (**a**) HER3+/HR+ MCF7 and T47D cells and (**b**) HER3-negative MDA-MB-231 and BCX-010 cells as determined by soft agar assay. **c** Effects of HER3-DXd (10 nM) and patritumab (10 nM) on colony formation in MCF7 and T47D cells as determined by clonogenic assay. Bars show the mean ± SD. ns: not significant, **P* < 0.05, ***P* < 0.01, *****P* < 0.0001. In **a**, **b** (right panels), and **c**, the data are representative of triplicates from one of three independent experiments. In **b** (left panels), the data are representative of triplicates from one of two independent experiments.

**Supplementary Fig. S3: Top 30 targets identified on the basis of fraction affected and sensitivity index in HER3-DXd–treated MCF7 cells by RNAi screening.**

**
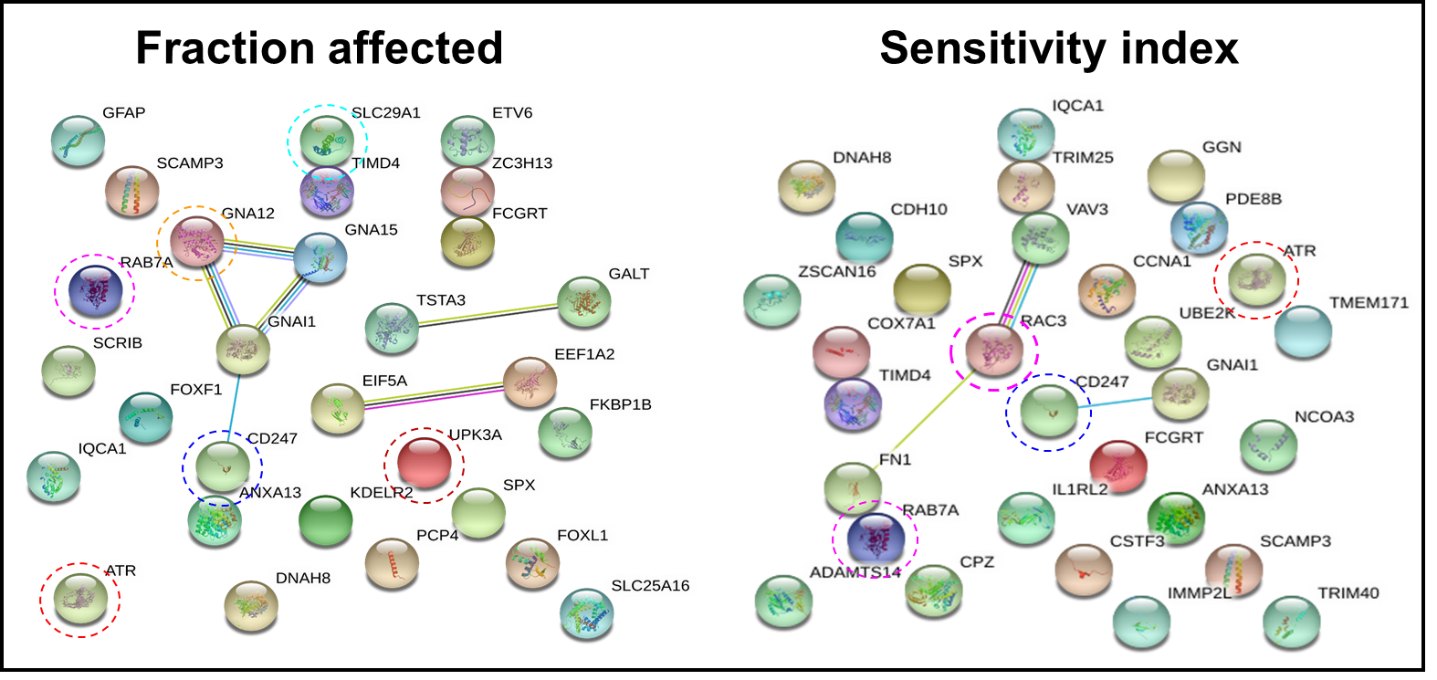
**

Top 30 targets identified on the basis of fraction affected and sensitivity index in HER3-DXd (20 nM)-treated MCF7 cells by RNAi screening using Ambion Silencer Select Human Genome siRNA Library V4 (64,752 siRNAs targeting 21,584 genes, with 3 unique siRNAs targeting each gene).


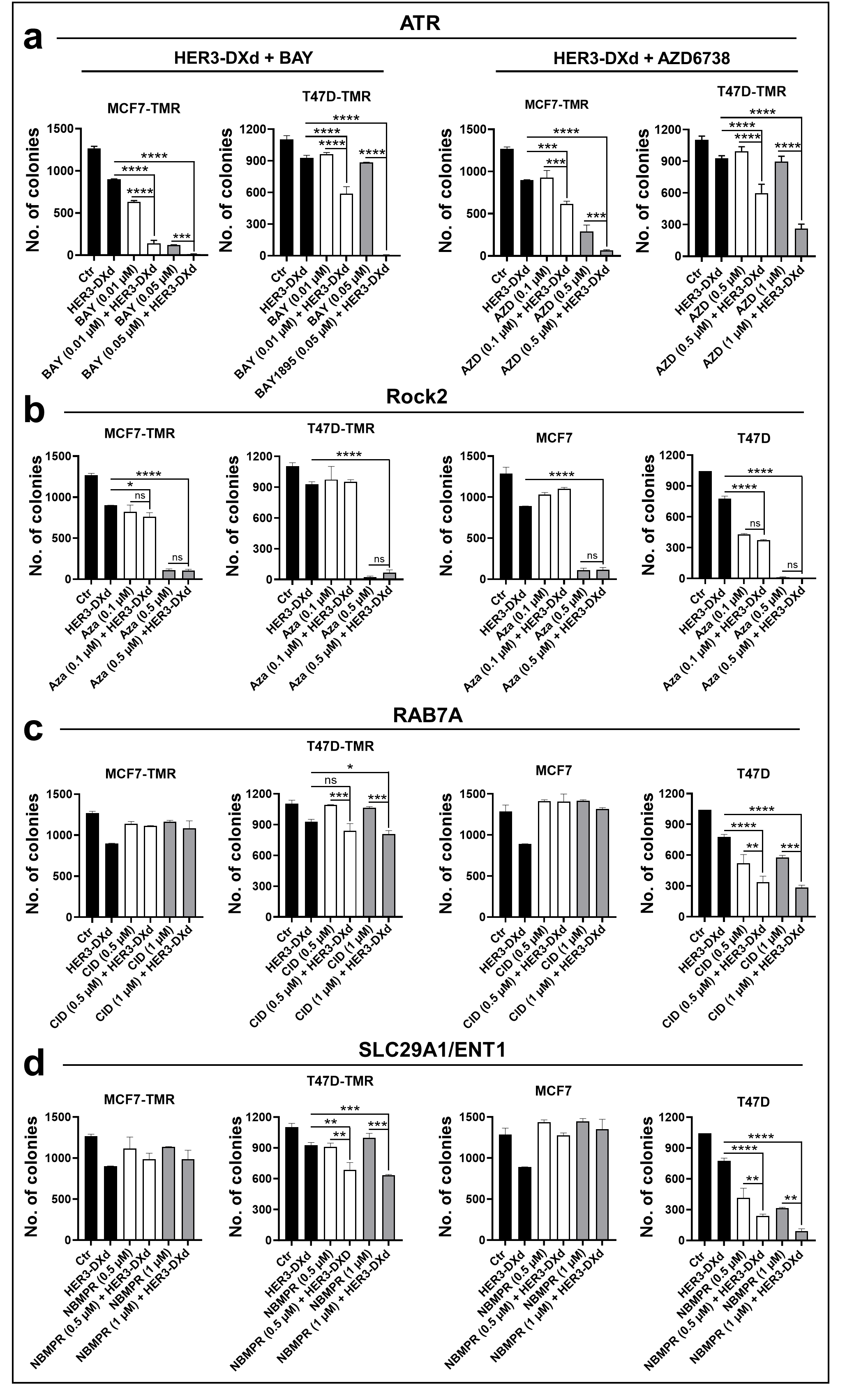
**Supplementary Fig. S4: The impact of targeting top candidates identified by RNAi screening on the growth-inhibiting activity of HER3-DXd in HER3+/HR+ MCF7, MCF7-TMR, T47D, and T47D-TMR cells.**

**a-d** Growth-inhibiting effects of HER3-DXd (5 nm) combined with (**a**) ATR inhibitors (AZD6738 [AZD] and BAY 1895344 [BAY]), (**b**) ROCK2 inhibitor azaindole 1 (Aza), (**c**) RAB7A inhibitor CID1067700 (CID), or (**d**) SLC29A1/ENT1 inhibitor NBMPR as determined by soft agar assay. Bars show the mean ± SD. ns: not significant, **P* < 0.05, ***P* < 0.01, ****P* < 0.001, *****P* < 0.0001. The data are representative of triplicates from one of two independent experiments.

**Supplementary Fig. S5: HER3-DXd inhibits MCF7 and T47D cell growth through DXd, and targeting ATR enhances this effect.**

**
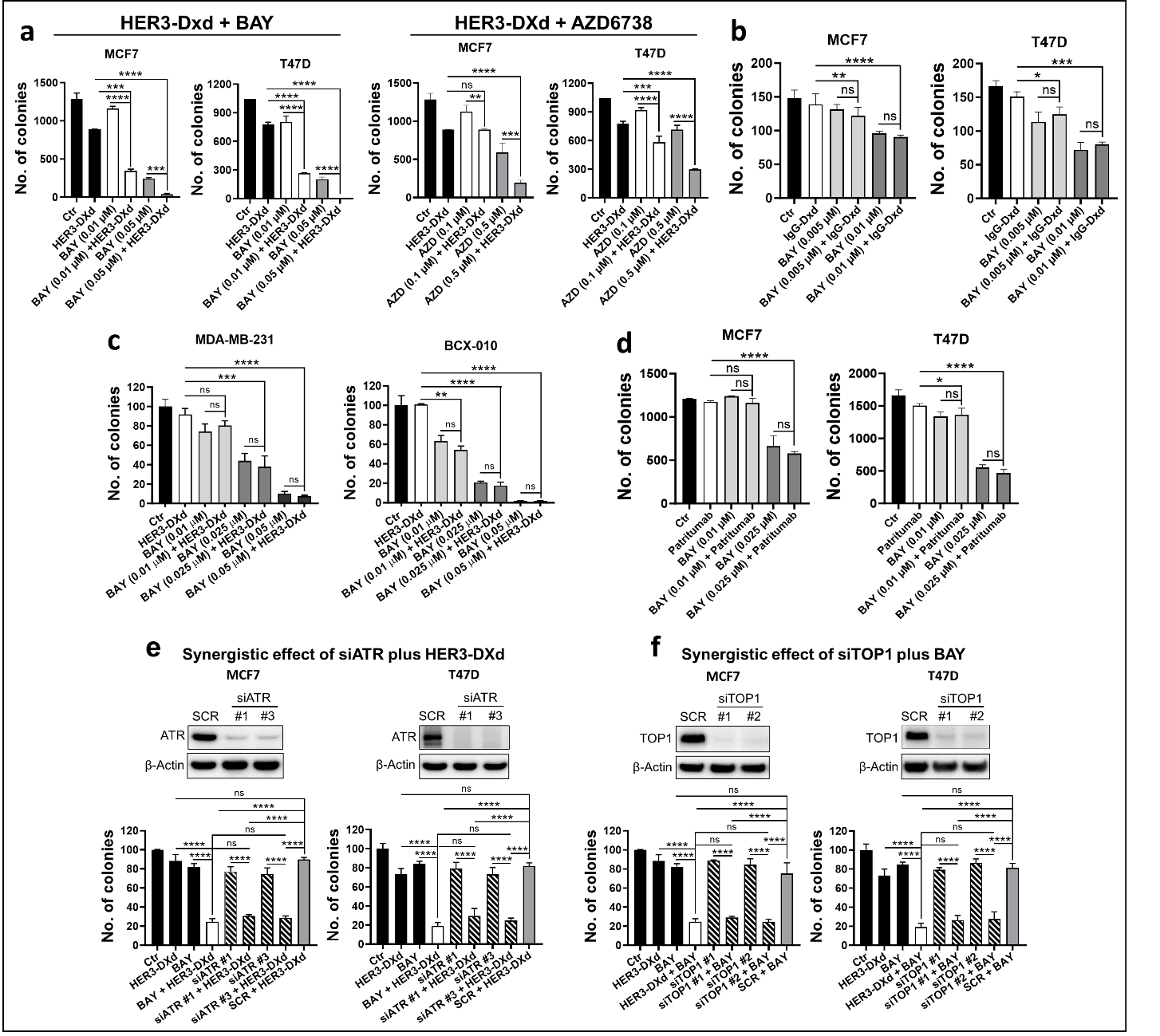
**

**a** Synergistic effects of HER3-DXd (5 nM) and ATR inhibitors (AZD6738 [AZD] and BAY 1895344 [BAY]) in MCF7 and T47D cells as determined by soft agar assay. **b** Effects of BAY plus IgG-DXd (5 nM) on colony formation in HER3+/HR+ MCF7 and T47D cells as determined by clonogenic assay. **c** Effects of BAY 1895344 (BAY) plus HER3-DXd (10 nM) on colony formation in HER3-negative MDA-MB-231 and BCX-010 cells as determined by clonogenic assay. **d** Effects of BAY plus patritumab (10 nM) on colony formation in HER3+/HR+ MCF7 and T47D cells as determined by soft agar assay. **e** and **f** HER3-DXd and BAY synergize by specifically targeting ATR and TOP1. **e** Knockdown of ATR using siRNA synergistically enhances the inhibiting effect of HER3-DXd (10 nM) on colony formation in HER3+/HR+ MCF7 and T47D cells as determined by clonogenic assay. **f** Knockdown of TOP1 using siRNA synergistically enhances the inhibiting effect of BAY (0.01 µM) on colony formation in HER3+/HR+ MCF7 and T47D cells as determined by clonogenic assay. Ctr, control (DMSO); SCR, scrambled control siRNA. Bars show the mean ± SD. ns: not significant, **P* < 0.05, ***P* < 0.01, ****P* < 0.001, *****P* < 0.0001. In **a-f**, the data are representative of triplicates from one of three independent experiments.

**Supplementary Fig. S6: HER3-DXd and BAY 1895344 synergize by inducing DNA damage, cell cycle arrest, and apoptosis.**


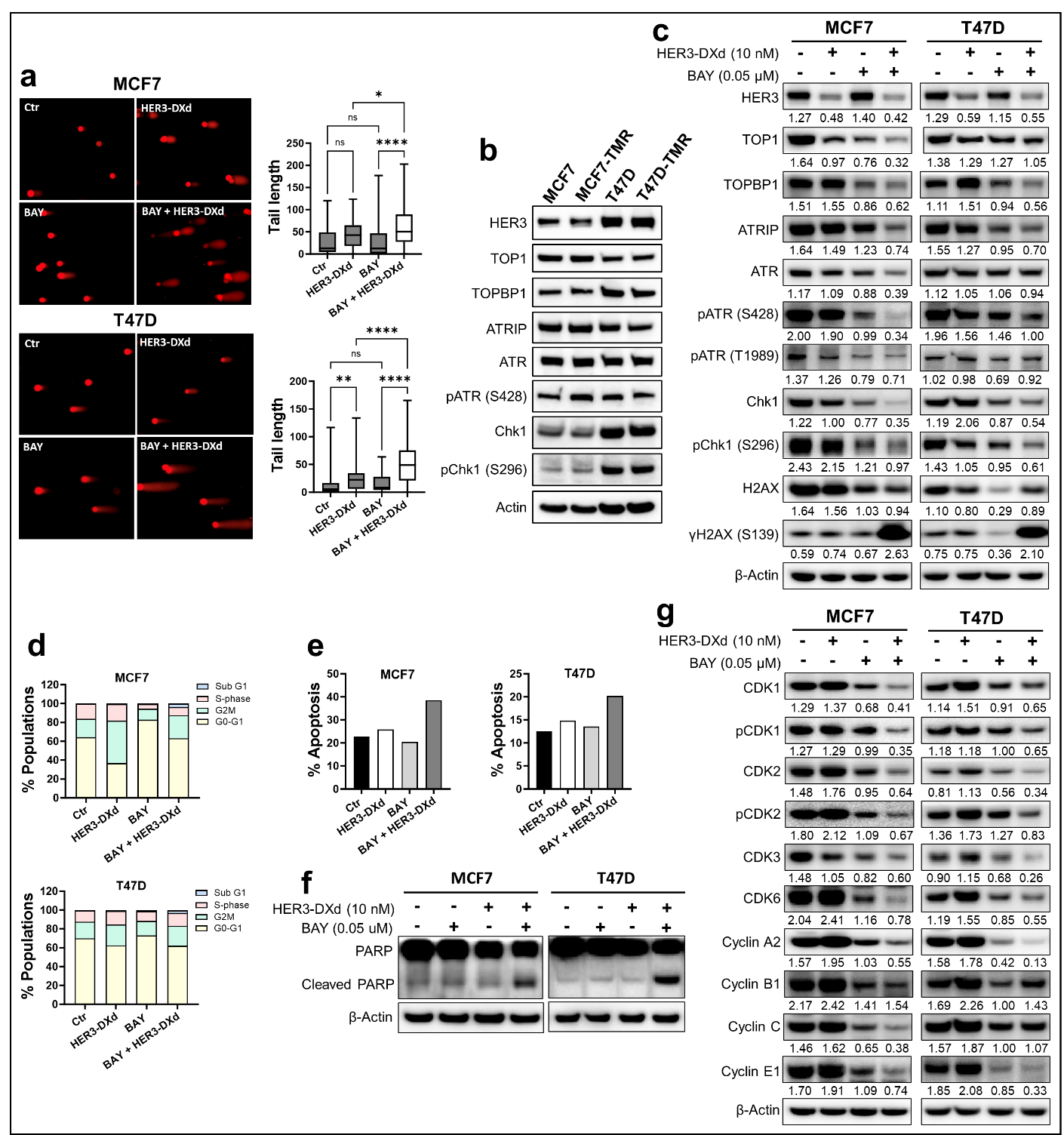


**a** Comet formation (left) and quantification of comets formed (right) in HER3+/HR+ MCF7 and T47D cells following treatment with DMSO (control; Ctr), HER3-DXd (10 nM), BAY 1895344 (BAY; 0.01 µM), or HER3-DXd plus BAY for 72 h. Bars show the mean ± SD. ns: not significant, **P* < 0.05, ***P* < 0.01, *****P* < 0.0001. **b** Baseline expression of proteins involved in DNA repair as analyzed by Western Blotting. **c** Expression of proteins involved in DNA repair following treatment with the Ctr, HER3-DXd, BAY, or HER3-DXd plus BAY for 48 h as analyzed by Western Blotting. **d** Effects on cell cycle progression following treatments with the Ctr, HER3-DXd (10 nM), BAY (0.01 µM), or HER3-DXd plus BAY for 72 h as analyzed by flow cytometry. **e** and **f** Apoptosis induction as analyzed by (**e**) flow cytometry and (**f**) Western blotting following treatment with the Ctr, HER3-DXd (10 nM), BAY (0.05 µM), or HER3-DXd plus BAY for 72 h. **g** Expression of proteins regulating cell cycle progression following treatment with the Ctr, HER3-DXd, BAY, or HER3-DXd plus BAY for 48 h as analyzed by Western blotting. In **b**, **c**, **f**, and **g**, β-Actin was used as a loading control. Band intensity of proteins is normalized to that of β-Actin. In **a**, **d**, and **e**, the data are representative of triplicates from one of three independent experiments. In **b**, **c**, **f**, and **g**, the data are representative of triplicates from one of two independent experiments.

**Supplementary Fig. S7: Effects of treatment on mouse body weights.**

**
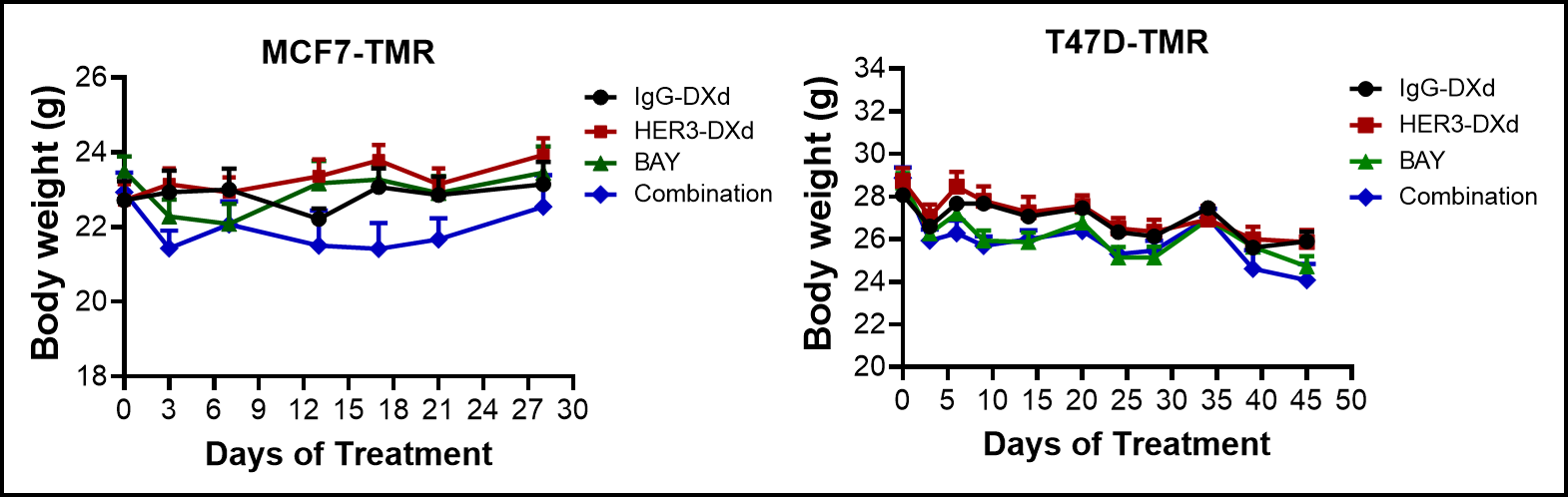
**

Effects of treatment with HER3-DXd, BAY 1895344 (BAY), and HER3-DXd plus BAY on mouse body weights in MCF7-TMR and T47D-TMR xenograft models.

**Supplementary Tables**

**Supplementary Table S1 Top candidates identified by high-through put RNAi screening using Ambion Silencer Select Human Genome siRNA Library V4 (64,752 siRNAs targeting 21,584 genes, with 3 unique siRNAs targeting 1 gene).**

| **Gene symbol** | **Sensitivity**  **index** | **Gene name(s)** | **Subcellular location** | **Type** |
| --- | --- | --- | --- | --- |
| ***ATR*** | 0.259 | ATR serine/threonine kinase,  ataxia telangiectasia and Rad3 related | Nucleus | Kinase |
| ***ROCK2*** | 0.236 | Rho-associated coiled-coil containing protein kinase 2 | Cytoplasm | Kinase |
| ***RAB7A*** | 0.194 | RAB7A,  member RAS oncogene family | Cytoplasm  Lysosomes | Enzyme |
| ***CD247*** | 0.188 | CD247 molecule,  T-cell surface glycoprotein CD3 zeta chain | Cytoplasm membrane | Transmembrane receptor |
| ***UPK3A*** | 0.176 | Uroplakin 3A | Transmembrane |  |
| ***SLC29A1* (formerly *ENT1*)** | 0.158 | Solute carrier family 29 member 1 | Cytoplasm membrane | Transporter |
| ***WNT7A*** | 0.153 | Wnt family member 7A | Extracellular Space | Cytokine |
